# Computationally Developed Sham Stimulation Protocol for Multichannel Desynchronizing Stimulation

**DOI:** 10.3389/fphys.2018.00512

**Published:** 2018-05-08

**Authors:** Magteld Zeitler, Peter A. Tass

**Affiliations:** ^1^Research Center Jülich, Institute for Neuroscience and Medicine, Brain and Behaviour (INM-7), Jülich, Germany; ^2^Department of Neurosurgery, Stanford University, Stanford, CA, United States

**Keywords:** sensory neurostimulation, non-invasive neuromodulation, coordinated reset, spike timing-dependent plasticity, desynchronization, anti-kindling, sham stimulation, placebo

## Abstract

A characteristic pattern of abnormal brain activity is abnormally strong neuronal synchronization, as found in several brain disorders, such as tinnitus, Parkinson's disease, and epilepsy. As observed in several diseases, different therapeutic interventions may induce a placebo effect that may be strong and hinder reliable clinical evaluations. Hence, to distinguish between specific, neuromodulation-induced effects and unspecific, placebo effects, it is important to mimic the therapeutic procedure as precisely as possibly, thereby providing controls that actually lack specific effects. Coordinated Reset (CR) stimulation has been developed to specifically counteract abnormally strong synchronization by desynchronization. CR is a spatio-temporally patterned multichannel stimulation which reduces the extent of coincident neuronal activity and aims at an anti-kindling, i.e., an unlearning of both synaptic connectivity and neuronal synchrony. Apart from acute desynchronizing effects, CR may cause sustained, long-lasting desynchronizing effects, as already demonstrated in pre-clinical and clinical proof of concept studies. In this computational study, we set out to computationally develop a sham stimulation protocol for multichannel desynchronizing stimulation. To this end, we compare acute effects and long-lasting effects of six different spatio-temporally patterned stimulation protocols, including three variants of CR, using a no-stimulation condition as additional control. This is to provide an inventory of different stimulation algorithms with similar fundamental stimulation parameters (e.g., mean stimulation rates) but qualitatively different acute and/or long-lasting effects. Stimulation protocols sharing basic parameters, but inducing nevertheless completely different or even no acute effects and/or after-effects, might serve as controls to validate the specific effects of particular desynchronizing protocols such as CR. In particular, based on our computational findings we propose a multichannel sham (i.e., inactive) stimulation protocol as control condition for phase 2 and phase 3 studies with desynchronizing multichannel stimulation techniques.

## Introduction

To establish a pharmacological therapy for clinical use, clinical trials are performed in humans that are typically classified into four phases (Friedman et al., 2015): First, in pre-clinical studies pharmacokinetic, toxicity, and efficacy are studied in non-human subjects. In phase I trials, so-called first in human-studies, safety and tolerability of a drug are investigated in healthy volunteers. Phase II trials aim to determine whether a drug can have any efficacy. More specifically, phase IIA trials typically aim at demonstrating clinical efficacy or biological activity (“proof of concept” studies), whereas phase IIB trials are dose-finding studies, performed to reveal optimum dose at which a drug has biological activity with minimal side-effects. Phase III trials investigate effectiveness and the clinical value of a new intervention in a larger patient group. In a randomized controlled trial the effect size of a new intervention is compared with state of the art treatment, if available. Finally, a phase IV trial is a postmarketing surveillance trial, performed e.g., to study whether any rare or long-term adverse effects occur within a much larger patient population and over longer time periods. Individual trials may actually comprise more than only one phase. For instance, there are combined phase I/II or phase II/III trials. Accordingly, given the different purpose of clinical trials, one may also distinguish between early phase studies and late phase trials (Friedman et al., 2015—see above). In principle, this 4-phase pattern also holds for medical technology, e.g., neuromodulation technologies.

Apart from investigating safety and tolerability, it is key to study whether a new therapeutic intervention is superior to pre-existing therapeutic options (Friedman et al., 2015). To this end, one has to take into account non-specific, placebo effects. A placebo effect is a psychobiological phenomenon that causes symptom relief after delivery of inert substances or other types of sham treatment, such as sham surgery or sham stimulation, in combination with verbal instructions suggesting clinical benefit (Price et al., 2008; Benedetti et al., 2011). Note, in clinical trials the terms placebo and sham are basically synonymous, while a placebo typically refers to an inactive substance used in pharmacological trials, whereas a sham stimulation/operation refers to a stimulation/operation without specific therapeutic effect (Price et al., 2008; Benedetti et al., 2011; Friedman et al., 2015). Real placebo effects go beyond spontaneous remission due to the natural history of a disease, regression to the mean induced by selection biases or expectation-related biases of patients and doctors (Benedetti, 2008a).

There are many different placebo effects, caused by different mechanisms and related to different types of interventions and different diseases (Benedetti, 2008b; Enck et al., 2008). Expectation, anxiety, reward, and different types of learning mechanisms may contribute to placebo effects (Benedetti et al., 2011). For instance, according to the Hawthorne effect, patients may simply improve because they are enrolled in a clinical trial (Last, 1983). Placebo treatments can decrease anxiety levels (Vase et al., 2005) and, in general, modulate emotions (Petrovic et al., 2002). Conversely, an inert substance combined with an instruction inducing negative expectations may cause a nocebo effect (Enck et al., 2008), e.g., an increase of pain (Colloca et al., 2008).

Placebo effects may actually be related to objective changes of brain action (Benedetti et al., 2011), e.g., release of endogenous dopamine (de la Fuente-Fernández et al., 2001), changes in brain glucose metabolism (Mayberg et al., 2002) or changes of the activity of specific neuronal populations (Benedetti et al., 2004).

Different types of learning mechanisms, e.g., conditioning, may play important roles in placebo mechanisms (Benedetti et al., 2011). For instance, administration of a placebo after delivery of active drugs may be more effective than placebo administration without the previous experience with the corresponding active drug (Sunshine et al., 1964; Batterman, 1966; Batterman and Lower, 1968; Laska and Sunshine, 1973; Amanzio and Benedetti, 1999; Colloca and Benedetti, 2006). Not only features related to a drug or therapeutic procedure may contribute to placebo-mediated clinical improvement, but also many other stimuli, related to medical environment, equipment, and personnel (Benedetti et al., 2011). From a clinical trials standpoint it is, hence, important to mimic the entire procedure of treatment delivery as well as possible, since even instructions and rituals of the treatment delivery and procedure may cause actual changes in brain activity that may be the same as those induced by the specific treatment (Benedetti et al., 2011). Accordingly, a vast majority (97%) of surveyed Parkinson's disease (PD) clinical researchers in the United States and Canada believe that even in the case of neurosurgical cell-based and gene therapies for PD double-blind, placebo-controlled trails have to be performed to assess safety and efficacy (Kim et al., 2005; Olanow, 2005). Ninety percentage of PD clinical researchers consider burr holes as justified for sham neurosurgery procedures, and a minority (<22%) even consider penetration of brain tissue to be justified for the neurosurgical sham control (Kim et al., 2005; Olanow, 2005). Hence, even in the case of clinical trials performed according to highest quality standards, e.g., in the field of deep brain stimulation (DBS) (Schuepbach et al., 2013), the comparison between qualitatively different therapeutic regimes, e.g., invasive neuromodulation plus medication vs. medication only, caused debates on whether the study design could reliably rule out placebo effects (Schüpbach et al., 2014).

There is a variety of strategies for the development of sham stimulation protocols. For instance, in the context of transcranial current stimulation a number of studies were devoted to the development of appropriate sham stimulation protocols, since current flow can elicit tingling or itching skin sensations, where different transcranial electrical stimulation methods have different cutaneous perception thresholds (Ambrus et al., 2010). In a comparative transcranial electrical stimulation study, a short-duration active protocol was used as sham, where the active stimulation was turned on only for a brief period, during which stimulation-related unwanted effects/perceptions were elicited (Inukai et al., 2016). Accordingly, the dose should be insufficient, but the patient should get the impression of receiving stimulation. Alternatively, off-target stimulation strategies were developed. In that case, the patient perceives stimuli and/or side effects thereof, but stimulation is directed to targets putatively rendering stimulation ineffective. For instance, for a sham condition for transcranial direct current stimulation a current configuration was chosen such that the current primarily traversed across the scalp, through adjacent pairs of electrodes of opposite polarity, in this way sparing cortical tissue (Richardson et al., 2014; Garnett and den Ouden, 2015). Another off-target stimulation sham strategy is used in the field of tinnitus, where stimulation tones are delivered at sufficiently detuned pitch compared to the tonal tinnitus, putatively activating brain sites sufficiently remote from the brain regions engaged in the tinnitus-related abnormal neuronal synchrony (Tass et al., 2012a; Adamchic et al, 2017). In contrast, we here consider the situation when clinical constraints are not permitting an off-target stimulation for sham purposes. We hypothesize that an appropriate stimulation pattern may render stimulation ineffective, although the single stimuli are delivered to target sites.

This computational study is dedicated to the development of sham stimulation protocols for desynchronizing multi-channel stimulation techniques, specifically coordinated reset (CR) stimulation (Tass, 2003a). The latter was computationally designed to specifically antagonize abnormal neuronal synchrony by desynchronization (Tass, 2003a,b). To this end, sequences of stimuli are administered to different neuronal sub-populations engaged in abnormal neuronal synchronization (Tass, 2003a). In computational studies it was shown that in the presence of spike-timing-dependent plasticity (STDP) (Gerstner et al., 1996; Markram et al., 1997; Bi and Poo, 1998) CR stimulation may have long-lasting, sustained effects (Tass and Majtanik, 2006; Hauptmann and Tass, 2007; Popovych and Tass, 2012). This anti-kindling effect (Tass and Majtanik, 2006) is caused by a CR-induced reduction of the rate of coincidences which, in turn, induces a decrease of synaptic weights, ultimately shifting the stimulated network from an attractor with abnormal synaptic connectivity and abnormal neuronal synchrony to an attractor with weak connectivity and synchrony (Tass and Majtanik, 2006; Hauptmann and Tass, 2007; Popovych and Tass, 2012).

Abnormal neuronal synchronization was found in a number of brain diseases, e.g., Parkinson's disease (Lenz et al., 1994; Nini et al., 1995; Hammond et al., 2007), tinnitus (Ochi and Eggermont, 1997; Llinas et al., 1999; Weisz et al., 2005; Eggermont and Tass, 2015), migraine (Angelini et al., 2004; Bjørk and Sand, 2008). Standard high-frequency (HF) DBS is the standard treatment of medically refractory movement disorders, such as PD (Benabid et al., 1991; Krack et al., 2003; Deuschl et al., 2006). Standard HF DBS only has acute clinical (Temperli et al., 2003) and acute electrophysiological (Kühn et al., 2008; Bronte-Stewart et al., 2009) effects, which are present only during stimulation and vanish after cessation of stimulation. In contrast, in parkinsonian nonhuman primates it was shown that electrical CR-DBS of the subthalamic nucleus (STN) has sustained, long-lasting after-effects on motor function (Tass, 2003b; Wang et al., 2016). Analogously, cumulative and lasting after-effects of electrical CR-DBS of the STN were also observed in PD patients (Adamchic et al., 2014).

For the clinical development, in particular, of non-invasive applications of CR stimulation (Popovych and Tass, 2012), such as acoustic CR stimulation for tinnitus (Tass et al., 2012a) or vibrotactile stimulation for PD (Tass, 2017; Syrkin-Nikolau et al., 2018), it is key to compare the effects of CR stimulation with an appropriate sham stimulation protocol in phase II and phase III clinical trials. The sham stimulation protocol should be reasonably similar to the CR stimulation pattern, to prevent patients from being able to distinguish between actual treatment and control. Accordingly, performing double-blind, placebo-controlled trails for non-invasive, sensory multichannel stimulation therapies requires multichannel sham stimulation protocols.

We here computationally develop a multichannel sham stimulation protocol. To this end, we investigate the anti-kindling effect of several multichannel stimulation protocols that share basic features with CR stimulation. We apply the different stimulation protocols to a one-dimensional computational network model with spiking neurons and study the stimulation effects at different levels, ranging from the macroscopic network level, via subpopulations down to the single neuron level. We obtain an inventory of qualitatively different stimulation effects elicited by the different stimulation protocols. Intriguingly, we found an inert stimulation protocol which caused only weak acute and hardly any long-lasting effects. The latter is a potential sham candidate to be tested for clinical studies in the context of desynchronizing sensory multichannel stimulation techniques.

## Materials and methods

In this section, we describe the equations used to model the dynamics of our one-dimensional neuronal network, the plasticity of the synapses, and the different stimulation protocols as well as the data analysis methods.

### Neuronal network

The model we use is a one-dimensional ring composed of *N* spiking Hodgkin-Huxley neurons which interact via strong excitatory short-range and weak inhibitory long-range synapses (Popovych and Tass, 2012). The membrane potential *V*_*i*_ of the *i*-th neuron (*i* = 1: *N*) is given by:

(1) $$\begin{array}{l} {C\dot{V}}_{i}=I_{i}-g_{Na}m_{i}^{3}h_{i}\left(V_{i}-V_{Na}\right)\\ -g_{k}n_{i}^{4}\left(V_{i}-V_{K}\right)-g_{l}\left(V_{i}-V_{l}\right)+S_{i}+F_{i},\\ {ẋ}_{i}=\alpha_{x}\left(V_{i}\right)\left(1-x_{i}\right)-\beta_{x}\left(V_{i}\right)x_{i},\\ {ṡ}_{i}=\frac{0.5\left(1-s_{i}\right)}{1+exp\left[-\left(V_{i}+5\right)/12\right]}-2s_{i}, \end{array}$$

where *C* denotes the membrane capacitance, the injected constant currents *I*_*i*_ are uniformly distributed (*I*_*i*_ϵ[*I*_0_ − Δ_*I*_, *I*_0_ + Δ_*I*_]) and *F*_*i*_ represents the current induced by an external stimulation signal (see section Simulation Details for more details). The voltage-dependent rate constants α_*x*_ and β_*x*_ of the time-varying ion gate variables *x* ϵ {*m, n, h*} are given by α_*m*_(*V*) = (0.1*V* + 4)/[1−*exp*(−0.1*V*−4)], β_*m*_(*V*) = 4 *exp*[(−*V*−65)/18], α_*h*_(*V*) = 0.07 *exp*[(−*V*−65)/20], β_*h*_(*V*) = 1/[1 + *exp*(−0.1*V*−3.5)], α_*n*_(*V*) = (0.01*V* + 0.55)/[1−*exp*(−0.1*V*−5.5)], and β_*n*_(*V*) = 0.125 *exp*[(−*V*−65)/80].

The coupling term *S*_*i*_ in Equation (1) stands for the weighted ensemble average of all postsynaptic currents received by neuron *i* from the other neurons in the network and can be given in terms of the synaptic variable *s*_*j*_ as:

(2) $$\begin{array}{l} {\;S}_{i}=N^{-1}\sum_{j=1}^{N}\left(V_{r,j}-V_{i}\right)c_{ij}|M_{ij}|s_{j}, \end{array}$$

where *N* is the number of neurons within the network, *V*_*r,j*_the reversal potential of the synaptic coupling between neurons *j* and *i* and *c*_*ij*_ is the synaptic coupling from neuron *j* to neuron *i. M*_*ij*_ has the form of a Mexican hat (Wilson and Cowan, 1973; Dominguez et al., 2006; de la Rocha et al., 2008) and determines the type of the neuronal connection between neurons *i* and *j*: *M*_*ij*_ < 0 represents an inhibitory coupling, *M*_*ij*_ > 0 an excitatory coupling. The value of *M*_*ij*_ determines the distance dependent maximal strength between those neurons:

(3) $$\begin{array}{l} M_{ij}=\left(1-d_{ij}^{2}/\sigma_{1}^{2}\right)\;exp\left(-d_{ij}^{2}/\left(2\sigma_{2}^{2}\right)\right) \end{array}$$

with σ_1_ = 3.5 and σ_2_ = 2.0 as in Popovych and Tass (2012), and *d*_*ij*_ = *d* · *min*(|*i*−*j*|, *N*−|*i*−*j*|) is the shortest distance between neurons *i* and *j*. To avoid boundary effects, the neurons form a one-dimensional ring. Therefore, the shortest distance between the neurons with indices 1 and *N* is 1 instead of *N*−1. The lattice distance between two adjacent neurons is given by:

(4) $$\begin{array}{l} d=\;d_{0}/\left(N-1\right) \end{array}$$

with *d*_0_ the length of the neuronal chain.

Values used in this study are *N* = 200, C = 1 μF/cm^2^, maximum conductance per unit area for the sodium, potassium and leak currents *g*_*Na*_ = 120 mS/cm^2^, *g*_*K*_ = 36 mS/cm^2^, *g*_*l*_ = 0.3 mS/cm^2^, sodium, potassium, and leak reversal potentials *V*_*Na*_ = 50 mV, *V*_*K*_ = −77 mV, and *V*_*l*_ = −54.4 mV, reversal potential for excitatory respectively inhibitory coupling *V*_*r,j*_ = 20 mV respectively−40 mV. For the constant injected current we used *I*_0_ = 11.0 μA/cm^2^ and _*I*_ = 0.45 μA/cm^2^. The length of the neuronal chain is defined as *d*_0_ = 10.

### Spike timing-dependent plasticity

The dynamical synaptic weights *c*_*ij*_ are influenced by the precise timing of the pre- and postsynaptic spikes and are updated in an event-based manner every time a neuron spikes. This is realized by adding δ · Δ*c*_*ij*_ to the excitatory and −δ · Δ*c*_*ij*_ to the inhibitory synaptic weights *c*_*ij*_ with learning rate δ > 0 every time neuron *i* or *j* spikes. According to the spike timing-dependent plasticity (STDP) rule (Bi and Poo, 1998) the change in synaptic weight is given by (Popovych and Tass, 2012):

(5) $$\begin{array}{l} \Delta c_{ij}=\{\begin{array}{c} \beta_{1}e^{\frac{-\Delta t_{ij}}{\gamma_{1\;}\tau }},\;\Delta t_{ij}\geq 0\\ \beta_{2}\frac{{\Delta t}_{ij}}{\tau }e^{\frac{\Delta t_{ij}}{\gamma_{2}\;\tau }},\;\Delta t_{ij}<0 \end{array} \end{array}$$

with Δ*t*_*ij*_ = *t*_*i*_ − *t*_*j*_ and *t*_*i*_ is the spike time of the postsynaptic neuron *i* and *t*_*j*_ the spike time of the pre-synaptic neuron *j*. Synaptic weights are restricted to the interval *c*_*ij*_ ∈ [0, 1] mS/cm^2^ to avoid unbounded strengthening and weakening. Other values used in relation to the STDP learning rule are as in Popovych and Tass (2012): β_1_ = 1, β_2_ = 16, γ_1_ = 0.12, γ_2_ = 0.15, τ = 14 ms, and δ = 0.002. With these parameter values, the plastic neuronal network under study is multistable, comprising stable desynchronized and stable synchronized states (Popovych and Tass, 2012).

### External stimulation

The aim of this study is to compare the effects of different stimulation algorithms on the neuronal connectivity as well as on the synchronization of the neuronal activity. In this section, we describe six different stimulation protocols as well as a no-stimulation control protocol to investigate the influence of the different stimulation protocols on the connectivity and synchronization.

#### Stimulation implemented in the model

Each stimulation onset induces single brief excitatory post-synaptic currents, with spatial spread in the network given by a quadratic spatial decay profile:

(6) $$\begin{array}{l} D\left(i,x_{k}\right)=\frac{1}{1+d^{2}{\left(i-x_{k}\right)}^{2}/\sigma_{d}^{2}} \end{array}$$

where *d* is the lattice distance between adjacent neurons (Equation 4), *i*−*x*_*k*_ the difference in index of neuron *i* and index *x*_*k*_ of the neuron at stimulation site *k*, and σ_*d*_ the spatial decay rate of the stimulation current (Popovych and Tass, 2012).

The total stimulation current induced in neuron *i* is given by:

(7) $$\begin{array}{l} F_{i}=\left[V_{r}-V_{i}\left(t\right)\right]\cdot K\sum_{k=1}^{N_{s}}D\left(i,x_{k}\right)G_{s,k}\left(t\right) \end{array}$$

with the excitatory reversal potential *V*_*r*_ = 20 mV, *V*_*i*_(*t*) the membrane potential of neuron *i* (Equation 1), *K* the stimulation intensity and *D* the spatial decay profile (Equation 6). *G*_*s,k*_ is the stimulation (at stimulation site *k*) evoked time-dependent normalized conductance of the post-synaptic membranes defined by α-functions. Since in this study the minimal time difference between two stimulation onsets within the network is not restricted to *T*_*s*_/*N*_*s*_ = 4 ms, we have adapted *G*_*s*_ from Popovych and Tass (2012) by allowing a summation of two stimulation evoked time-dependent normalized conductances of the post-synaptic membranes if the two stimulations occur within a certain time interval. We have set this time interval to 2 · *T*_*s*_/*N*_*s*_ = 8 ms, since at 8 ms the value of the α-function is marginal (only 0.02% of the peak value). Our adapted stimulation (at stim site *k*) evoked time-dependent normalized conductance of the post-synaptic membranes is now given by:

(8) $$\begin{array}{l} G_{s,k}\left(t\right)=\{\begin{array}{ll} \frac{t-t_{k}^{n}}{\tau }exp\left[-\frac{t-t_{k}^{n}}{\tau }\right], & t_{k}^{n}\leq t\leq \min(t_{k}^{n}+\frac{T_{s}}{2},t_{k}^{n+1})\\ \frac{t-t_{k}^{n}}{\tau }exp\left[-\frac{t-t_{k}^{n}}{\tau }\right]+\frac{t-t_{k}^{n+1}}{\tau }exp\left[-\frac{t-t_{k}^{n+1}}{\tau }\right], & t_{k}^{n+1}\leq t\leq t_{k}^{n}+T_{s}/2\;\\ 0, & otherwise \end{array} \end{array}$$

Here $t_{k}^{n}$ is the onset of the *n*th activation of the *k*th stimulus site, τ = *T*_*s*_/(6*N*_*s*_) represents the time-to-peak of *G*_*s,k*_, and min(*t*_1_, *t*_2_) is the minimum value of *t*_1_, *t*_2_ and thus represents the earliest time event of *t*_1_ and *t*_2_.

#### Stimulation signal features

As a control condition we use the situation where no stimulation signal is applied and thus no stimulation current is delivered to the *N* neurons: *F*_*i*_ = 0 for all *i* ∈ {1, .., *N*}. We denote the control condition as *no stimulation (no-stim) protocol*. The other, active stimulation protocols last 128 s and consist of stimulation ON and stimulation OFF cycles (briefly *ON- and OFF-cycles*), of duration *T*_*s*_ each. *N*_*s*_ equidistantly spaced stimulation sites are activated exactly once during one ON-cycle. After three consecutive ON-cycles, two OFF-cycles follow, before the next three ON-cycles take place. At the end of the stimulation period (briefly *stim-on period*) each stimulation site will have been activated exactly 4,800 times (=4,800 ON-cycles). In this study, each cycle lasts for *T*_*s*_ = 16 ms, and for all stimulation signals the same *N*_*s*_ = 4 equidistantly spaced stimulation sites are activated. The four stimulation sites are located at the neurons with index 25, 75, 125, and 175.

**Figure 1:**
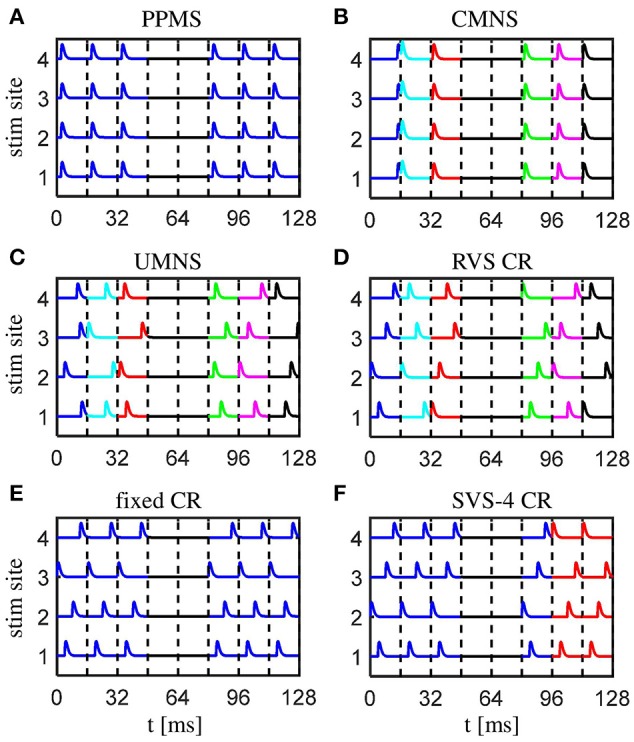
Schematic of the spatio-temporally patterned stimulation protocols as used in this study. **(A)** The purely period multichannel stimulation (PPMS) simultaneously activates all stimulation sites at the same time instance within each stimulation ON-cycle. **(B)** The correlated multichannel noisy stimulation (CMNS) activates all stimulation sites simultaneously, but at different, random time instances, equally distributed within each stimulation ON-cycle. **(C)** The uncorrelated multichannel noisy stimulation (UMNS) activates the stimulation sites sequentially in a random order at different time instances within different stimulation ON-cycles. Stimulation onsets are equally distributed within each stimulation ON-cycle, where the random processes for the different channels are uncorrelated. **(D)** The rapidly varying sequences (RVS) CR stimulation activates the stimulation sites in a random temporal order at times *n* · *T*_*s*_/4 (*n* ∈ {0, 1, 2, 3}) within each stimulation ON-cycles. **(E)** The fixed CR stimulation approach employs a fixed sequence during the entire CR-on period, and stimulation onsets times within the stimulation ON-cycles are at multiples of *n* · *T*_*s*_/4 (*n* ∈ {0, 1, 2, 3}). **(F)** In this example, for the sake of illustration, the slowly varying sequences (SVS) CR stimulation repeats the first sequence four times, before a new sequence is drawn. Different stimulation sequences are randomly drawn with equal probability. For the remainder of this study each sequence is repeated 100 times and not just four times (SVS-100 instead of SVS-4). Stimulation onsets are as for the RVS CR at *n* · *T*_*s*_/4 (*n* ∈ {0, 1, 2, 3}) within the stimulation ON-cycles. All stimulation protocols have four equidistantly distributed stimulation sites, stimulation ON- and OFF- cycles of 16 ms, a total stimulation on period of 128 s, in which three ON-cycles are alternated by two OFF-cycles. No stimulation is applied during OFF-cycles. Stimulation ON- and OFF-cycles are separated by dashed vertical lines. A change of color indicates a change of the pattern of stimulus onsets compared to the previous ON-cycle.

The first of the six stimulation signal approaches is the *purely periodic multichannel stimulation (PPMS)*. The first stimulation onset of the *k*-th stimulation site, $t_{k}^{1}$, is drawn randomly (with equal distribution within an ON-cycle), the next stimulation onsets occur exactly a multiple of *T*_*s*_ later: $t_{k}^{n}=\;t_{k}^{1}+n\cdot T_{s}$ (with *k* ∈ {1, 2, …, *N*_*s*_} and *n* ∈ ℕ). Another feature of this PPMS approach is that all four stimulation sites are activated simultaneously (see Figure 1A), which implies that $t_{k}^{n}=t_{1}^{n}$ for all stimulation sites *k*.

The *correlated multichannel noisy stimulation (CMNS)* activates, like the PPMS, all four stimulation sites simultaneously, but the stimulation onsets are no longer periodic, but rather noisy: For each ON-cycle the stimulation onset is drawn randomly, with equal probability within the ON-cycle (Figure 1B). In this case, the four stimulation sites are also active simultaneously: $t_{k}^{n}=t_{1}^{n}$ for all stimulation sites *k*.

For the *uncorrelated multichannel noisy stimulation (UMNS)* the stimulation onsets $t_{k}^{n}$ are determined for each stimulation site *k* separately. To this end, for each stimulation site *k* and ON-cycle *n* the stimulation onset $t_{k}^{n}$ is drawn randomly, with equal distribution within the *n*-th ON-cycle. The random processes that generate the stimulation onsets $t_{k}^{n}$ for the different stimulation sites *k* ∈ {1, 2, …, *N*_*s*_} are completely uncorrelated between stimulation sites and, hence, typically do not coincide (Figure 1C).

The other three stimulation protocols are different variants of coordinated reset (CR) stimulation (Tass, 2003a,b). For CR stimulation, within each ON-cycle the activations of the *N*_*s*_ = 4 different stimulation sites are equidistantly spaced in time, with a time shift of *T*_*s*_/*N*_*s*_ (Figures 1D–F). The different stimulation onsets are at the beginning of the ON-cycle, 4, 8, and 12 ms later. The order in which the stimulation sites are activated exactly once during an ON-cycle is called a stimulation sequence, briefly *sequence*. For the RVS CR the sequence randomly changes for one ON-cycle to the next. In contrast, for the fixed CR the same sequence is maintained for all ON-cycles (Figure 1E). For the slowly varying sequences (SVS-*n*) CR a sequence is applied during *n* ON-cycles, before randomly switching to another sequence which is, in turn, used for the next *n* ON-cycles etc (Zeitler and Tass, 2015). Figure 1F illustrates SVS-4 CR, where each sequence is repeated four times before another sequence is drawn randomly. In the SVS CR protocols of this study we will only apply *n* = 100 consecutive repetitions of a sequence before we draw the next sequence. Therefore, we will use the term SVS instead of SVS-100 CR for the remainder of this work.

### Simulation details

A simulation contains four different simulation periods: first an initializing period of 2 s, then a 60 s period with STDP and without external stimulation (denoted as *STDP-only period*) which is followed by a stim-on period of 128 s in which the network receives an external stimulation and after withdrawal of the stimulation follows a stimulation off period (denoted as *stim-off period*) of 128 s. During the initializing period - the only period without STDP - a network is built by drawing random numbers for each of the *N* = 200 neurons from uniform distributions for the injected constant current *I*_*i*_ ∈ [*I*_0_ − Δ_*I*_, *I*_0_ + Δ_*I*_], for the membrane potential *V*_*i*_ ∈ [−65, +5] ms, for the time-varying ion gate variables *x*_*i*_ ∈ [0, 1] and the synaptic variable *s*_*i*_ ∈ [0, 1]. The initial synaptic weights *c*_*ij*_ are drawn from a Gaussian distribution with mean 0.5 μA/cm^2^ and standard deviation 0.01 μA/cm^2^. During the initializing period the neuronal network evolves without influences of external stimulation signals or STDP.

For this selected initial distribution of the synaptic weights, during the STDP-only period the network can develop into a strongly connected network with strongly synchronized neuronal activity. The time at which the first stimulation signal is delivered to the network is defined as *t* = 0 s. During the stim-on period the stimulation signals are applied to the network as described in section Stimulation Implemented in the Model. After 128 s no stimulation signals are applied anymore and the evolution of the network is monitored for another 128s (stim-off period). After going through this whole process, the procedure is repeated from *t* = 0 s on for a different stimulation intensity, *K*, and/or for a different stimulation signal approach.

From previous studies (Zeitler and Tass, 2015, 2016) we know that different initial network conditions and different stimulation signal realizations have an influence on the anti-kindling effects. Therefore, we draw eleven different sets of initial values from the distributions as described above at the start of the initializing period. For each of these eleven different initial network conditions we generate one realization for each stimulation signal approach. In the remainder of this study the combination of a stimulation signal realization with one set of initial network conditions is referred to as *sample*. All simulations were executed in Matlab R2007a. The differential equations were solved by the built-in function ODE45 with a relative tolerance of 10^−5^.

### Data analysis

In this computational study we came up with a larger set of acute and long-lasting effects of different stimulation protocols applied to the plastic neural network as described in section Neuronal Network. We are particularly interested in whether the different stimulation protocols might induce qualitatively different anti-kindling effects. In this section, we will discuss the methods used to investigate the neuronal connectivity at several levels, the synchronization and a quantification of the acute stimulation and acute after-effects, as well as the phase resetting and entrainment induced by the different stimulation protocols. Matlab R2015a was used for the data analysis and for plotting the results.

#### Connectivity

In this study, the synaptic weights can change according to the STDP-rule (see Equation 5). On the network level the dynamics of the synaptic connectivity is monitored by the synaptic weight averaged over all synapses within the network:

(10) $$\begin{array}{l} C_{av}\left(t\right)=N^{-2}\sum_{i=1}^{N}\sum_{j=1}^{N}sign\left(M_{ij}\right)c_{ij}\left(t\right),\; \end{array}$$

where *N* is the number of neurons within the network, *sign*(*M*_*ij*_) is negative for inhibitory synapses and positive for excitatory synapses (with *M*_*ij*_defined as in Equation 3), and *c*_*ij*_ is the synaptic coupling strength from neuron *j* to *i*. A decrease of *C*_*av*_ over time may indicate that there is mainly a decrease in the average excitatory synaptic weights or an increase in average inhibitory weights or a combination of both. To unravel the contributions of the excitatory and the inhibitory synaptic weights, we introduce the average excitatory synaptic weight

(11) $$\begin{array}{l} c_{EE}\left(t\right)=\;N_{EE}^{-2}\sum_{i=1}^{N}\sum_{j=1}^{N}{\left[sign\left(M_{ij}\right)\right]}_{+}c_{ij}\left(t\right),\; \end{array}$$

and the average inhibitory synaptic weight

(12) $$\begin{array}{l} c_{II}\left(t\right)=\;N_{II}^{-2}\sum_{i=1}^{N}\sum_{j=1}^{N}{\left[sign\left({-M}_{ij}\right)\right]}_{+}c_{ij}\left(t\right),\; \end{array}$$

where [*z*]_+_ stands for the half-wave rectification operation ([*z*]_+_ = *z* if *z* > 0 and [*z*]_+_ = 0 otherwise), *N*_*EE*_ is the number of excitatory synapses, and *N*_*II*_ is the number of inhibitory synapses within the whole neuronal network.

On the neuronal level the connectivity matrices are analyzed at the end of the stim-on and at the end of the stim-off period, since we are interested in the acute and the long-lasting effects. Instead of considering the *c*_*ij*_-values, we first multiplied each *c*_*ij*_-value by the sign-function of the Mexican Hat, *sign*(*M*_*ij*_) (see Equation 3 for *M*_*ij*_). This allows to recognize the type of each synapse in a color plot of the connectivity matrix (negative values for inhibitory and positive values for excitatory synapses). For each synapse eleven *c*_*ij*_-values exist since each stimulation protocol is applied at stimulation intensity *K* to eleven different initial networks. By determining the median and the inter-quartile-range (IQR) of these eleven *c*_*ij*_-values for all *i, j* ∈ {1, 2, …, *N*] we obtained a larger reduction of the amount of data. Unfortunately, due to the different initial network conditions the general connectivity pattern induced by a stimulation protocol does not straightforwardly reveal at which locations neurons are coupled mainly by bidirectional weak as opposed to unidirectional strong synapses.

To get a better idea of what happens due to a stimulation signal, we first sorted for each sample the *c*_*ij*_(*t*) values such that the strongest synaptic weight between two neurons *i* and *j* is placed in the lower right triangle at location (*i*′,*j*′) = (min(*i*,*j*), max(*i*,*j*)) and the weaker synaptic weight between those two neurons *i* and *j* in the upper left triangle of the connectivity matrix at location (*i*′,*j*′) = (max(*i*,*j*), min(*i*,*j*)). This is repeated for all combinations of two neurons. After that the result is multiplied by *sign*(*M*_*ij*_). We call this newly defined matrix the sorted connectivity matrix. After calculating the median of the eleven sorted connectivity matrices the general pattern is more evident for all stimulation protocols and the IQRs provide a clearer picture than for the unsorted connectivity matrices. By sorting the data like this, one can determine from the median if the synapses are in general strong in both directions or just in one direction or maybe even weak in both directions between two neurons. Furthermore, the IQR shows which type of synapses and at which spatial location the largest differences occur as an effect of the different samples. Note that the location (*i, j*) in this sorted connectivity matrix does not indicate that neuron *i* is the post-synaptic neuron and neuron *j* the pre-synaptic one as is the case for the unsorted connectivity matrices, but that if *i* < *j* (lower right triangle of the sorted matrix) than $c_{ij}^{sorted\;}=\max\left(c_{ij},c_{ji}\right)$ with *c*_*ij*_, *c*_*ji*_ elements of the unsorted connectivity matrix. For the upper left triangle of the sorted matrix where *i* > *j* this means that $c_{ij}^{sorted\;}=\min\left(c_{ij},c_{ji}\right)$.

#### Synchronization

The effect of the external stimulation signals on the strongly synchronized neuronal activity is investigated on the network level by the order parameter *R* (Haken, 1983; Kuramoto, 1984) defined by

(13) $$\begin{array}{l} R\left(t\right)exp\left[i\Phi \right]=N^{-1}\sum_{j=1}^{N}exp\left[i\varphi \left(t\right)\right] \end{array}$$

where Φ(t) is the circular mean phase of the entire group of *N* neurons in the network, and

(14) $$\begin{array}{l} \varphi_{j}\left(t\right)=2\pi \left(t-t_{j,m}\right)/\left(t_{j,m+1}-t_{j,m}\right)\text{ for }t_{j,m}\leq t<t_{j,m+1} \end{array}$$

is a linear approximation of the phase of neuron *j* between its *m*-th and (*m* + 1)-th spikes at spike times *t*_*j,m*_ and *t*_*j,m*+1_ (Rosenblum et al., 2001). The minimum value of *R* is zero and indicates a complete lack of in-phase synchronization, whereas its maximum value (*R* = 1) indicates perfect in-phase synchronization.

For our analysis, we have calculated *R*, Φ, and all φ_*j*_ at each ms of the stim-on and stim-off period. In case an *R*-value is shown at a certain time instance *t* the *R*-values of the preceding 5 s period are averaged and denoted as *R*_*av*_ at *t*.

On a mesoscopic level the amount of synchronization of the *k*-th subpopulation can be defined as

$$\begin{array}{l} {\bar{R}}_{k}^{pre}={<\;R_{k}>}_{last\;5\;s\;before\;stim-ON}\\ {\bar{R}}_{k}^{on}={<\;R_{k}>}_{last\;5\;s\;of\;stim-ON}\\ {\bar{R}}_{k}^{off}={<\;R_{k}>}_{last\;5\;s\;of\;stim-OFF} \end{array}$$

were *R*_*k*_ denotes the synchronization order parameter of the *k*-th subpopulation (*k* ∈ {1, 2, …, *N*_*s*_}) as determined by

(15) $$\begin{array}{l} R_{k}\left(t\right)exp\left[i\Phi_{k}\left(t\right)\right]=N_{k}^{-1}\sum_{j=1}^{N_{k}}exp\left[i\varphi_{j}\left(t\right)\right] \end{array}$$

with *N*_*k*_ = 49 the number of neurons within subpopulation *k*, and φ_*j*_(*t*) the linear approximation of the phase of neuron *j* in subpopulation *k* at time *t* as determined by Equation (14). Using this definition, we can determine the acute stimulation effect on the *k*-th subpopulation by

(16) $$\begin{array}{l} 1-\frac{{\bar{R}}_{k}^{on}}{{\bar{R}}_{k}^{pre}} \end{array}$$

and the acute after-effect on the *k*-th subpopulation by

(17) $$\begin{array}{l} 1-\frac{{\bar{R}}_{k}^{off}}{{\bar{R}}_{k}^{pre}} \end{array}$$

A negative outcome indicates a synchronizing effect, a zero outcome indicates that there is no acute stimulation effect or after-effect, respectively, and a positive outcome means a desynchronizing effect within the *k*-th subpopulation due to application of that particular stimulation protocol.

#### Stimulus-locked phase dynamics of a subpopulation

To shed more light on the mechanisms of the different stimulation protocols and reveal, e.g., phase resetting or entrainment processes, we investigate the stimulus-locked dynamics on a mesoscopic scale. We do this by considering subpopulations of neurons as given by their proximity to the *N*_*s*_ = 4 different stimulation sites located at neuron indices 25, 75, 125, and 175. This implies that each subpopulation *k* contains *N*_*k*_ = *N*/*N*_*s*_−1 = 49 neurons, since the subpopulations are separated by a neuron which has an equal distance to the stimulation sites in the two neighboring subpopulations, e.g., the neuron at index 50 has the same distance to the stimulation site at neuron index 25 as well as to the stimulation site at neuron index 75 and is therefore excluded from subpopulation 1 as well as from subpopulation 2. The mean phase Φ_*k*_(*t*) of subpopulation *k* (*k* ∈ {1, 2, …, *N*_*s*_}) is determined by Equation 15. We focus on the distribution of the stimulus-locked phase dynamics within a time window *W*of 32 ms before and up to 32 ms after each of the *L* stimulation onsets $t_{k}^{n}$ (*n* = 1, .., *L*) for each subpopulation *k* separately (Tass, 2003c)

(18) $$\begin{array}{l} {\left\{\Phi_{k}\left(t_{k}^{n}+\Delta t\right)\left(mod\;2\pi \right)\right\}}_{n=1,\ldots ,L}. \end{array}$$

These distributions of the stimulus-locked phase dynamics of a subpopulation will be displayed in color plots as a function of Δ*t* ∈ *W*. To quantify the amount of stimulus locking of the phase dynamics of subpopulation *k*, we use the resetting index *E*_*k*_(*t*) (Tass, 2003c; see also Tallon-Baudry et al., 1996) as given by

(19) $$\begin{array}{l} E_{k}\left(\Delta t\right)=|L^{-1}\sum_{n=1}^{L}exp\left[i\Phi_{k}\left(t_{k}^{n}+\Delta t\right)\right]|. \end{array}$$

In case of a phase entrainment (a permanent stimulus-locking of the phase dynamics) *E*_*k*_(Δ*t*) results in a uniform distribution throughout the window *W* = [−32, +32] ms. For a stimulus-induced phase reset, *E*_*k*_(Δ*t*) will be small in the period before stimulus onset (corresponding to a uniform phase distribution) and will increase after stimulus onset, reflecting the emergence of a unimodal phase distribution (Tallon-Baudry et al., 1996; Tass, 2003c). For a stimulus-locked disruption of a phase entrainment the phase distribution will be unimodal before stimulus onset and decrease due to stimulus onset. Since all our stimulation protocols have the same ratio of ON: OFF cycles, namely 3:2, we can split each set of the *L* stimulation onsets $t_{k}^{n}$ (*n* = 1, .., *L*) for each subpopulation *k* into three subsets. The first subset contains only the *L*/3 stimulation onsets $t_{k}^{n}$ (*n* = 1, 4, .., *L*−2) within the first of the three consecutive ON-cycles, the second one contains the *L*/3 stimulation onsets $t_{k}^{n}$ (*n* = 2, 5, .., *L*−1) during the middle of each block of three consecutive ON-cycles and the last subset contains the *L*/3 stimulation onsets $t_{k}^{n}$ (*n* = 3, 6, .., *L*/3) within the third ON-cycle of each three consecutive ON-cycles. By using these three subsets separately instead of the three subsets together, the corresponding three resetting indices can be determined in a similar way as in Equation 19. This detailed analysis shows how the phase reset and entrainment processes evolve depending on the rank order of the onset after the OFF-cycles.

#### Statistics

To test whether an increase of the median of the eleven *C*_*av*_ or *R*_*av*_ values obtained by stimulations with protocol A at stimulation intensity *K*_*A*_ compared to stimulation protocol B applied at *K*_*B*_ is statistically significant, we used the Matlab R2015a built-in left-sided Wilcoxon rank sum test with significance level α: [p, h] = ranksum(A,B, “alpha”,0.05, “tail,” “left,” “method,” “exact”). This test is equivalent to the Mann-Whitney U-test. The built-in right-sided Wilcoxon rank sum test tests if a decrease in medians is statistically significant. In this study, we use significance level α = 0.05 and *n*_*A*_ = *n*_*B*_ = 11 samples unless stated otherwise.

## Results

### Acute effects

In this section, we study the acute stimulation effects of the different spatio-temporally patterned stimulation protocols at *K* = 0.25 by comparing their effects on the connectivity as well as the synchronization at the end of the stim-on period (*t* = 128 s).

**Figure 2:**
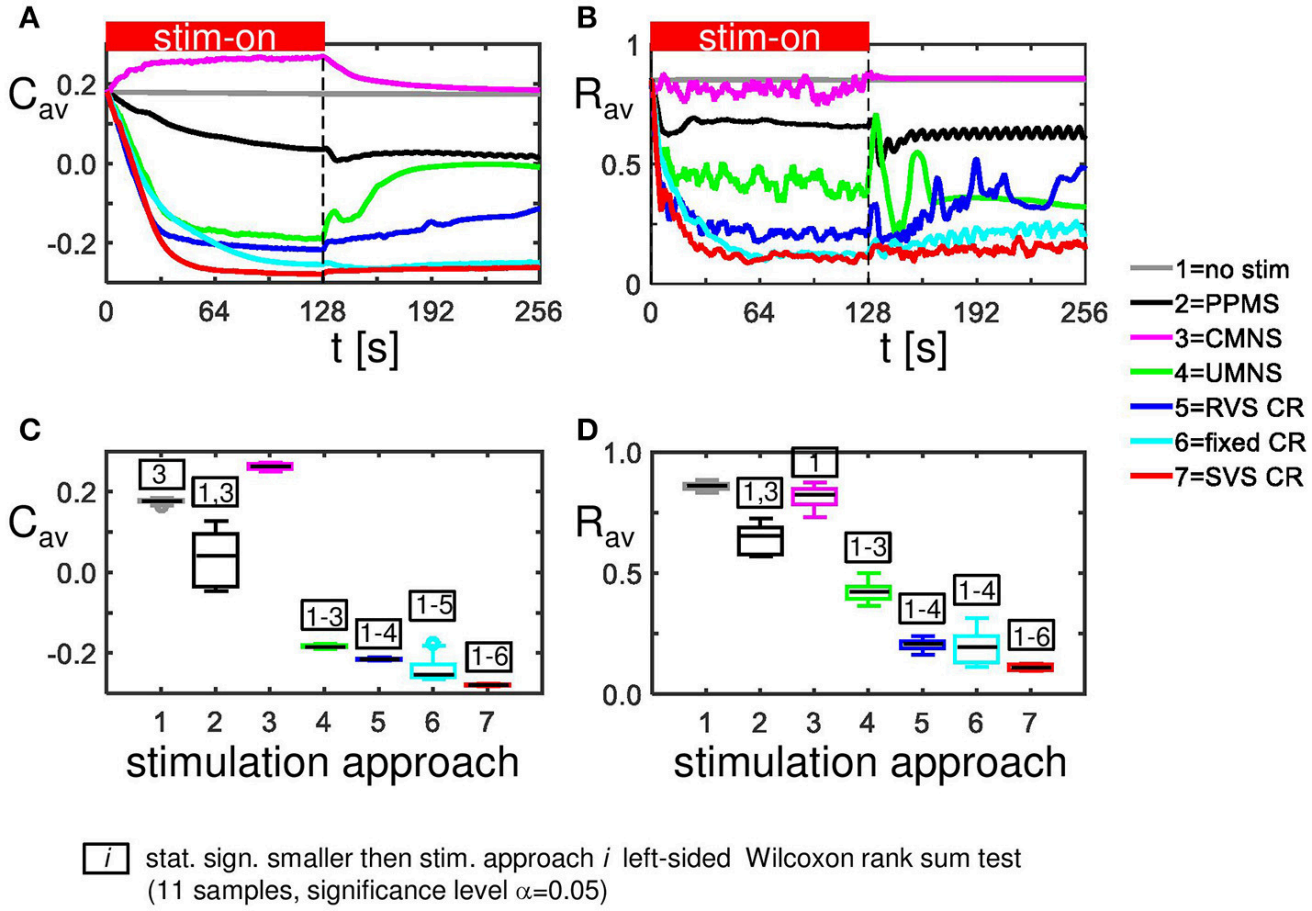
Acute and long-term effects depend on the stimulation protocol. **(A)** Stimulation effect on the connectivity on the population level. All stimulation protocols induce a decrease of the average synaptic weight *C*_*av*_, except the CMNS, which induces an increase during the stim-on period. This increase evolves to the initial value after withdrawal of the stimulation. **(B)** Stimulation effect on the synchronization of the population activity. All stimulation protocols induce a decrease of the amount of synchronization *R*_*av*_, except the CMNS, which basically only causes fluctuations during the stim-on period. These fluctuations vanish after stimulation withdrawal. In contrast, all other stimulation protocols cause a long-lasting desynchronization. All stimulation protocols are applied to the same initial network conditions and with stimulation intensity *K* = 0.25 during the stim-on period (*t* = 0–128 s). The red horizontal bar represents the stim-on period. No stimulation signals are delivered during the subsequent 128 s stim-off period. **(C)** Boxplots of *C*_*av*_ (*t* = 128 s) for different stimulation protocols show a statistically significant decrease compared to the no-stim approach except for the CMNS, which induces an increase of *C*_*av*_. **(D)** Boxplots of *R*_*av*_ (*t* = 128 s) show a statistically significantly desynchronization induced by the different stimulation protocols compared to the control condition (no-stim). Eleven samples (different combinations of initial network conditions and sequence orders) are used for each boxplot. The horizontal line within the box represents the median, the length of the box the IQR (middle 50%) and the whiskers below and above the box the first and last 25%. Outliers are defined as 1.5 times the length of the box below or above the box and are represented by open circles. *P*-values of the left-sided Wilcoxon rank sum test are given in Supplementary Table 1.

External stimulation signals can change the network's connectivity (due to STDP) and the amount of synchronized neuronal activity. The control signal (no-stim) has no influence on the average synaptic weight, *C*_*av*_ (Figure 2A) and on the synchronization of the population activity (shown by the order parameter *R*_*av*_ in Figure 2B). For the same initial network conditions the other stimulation signals applied at intensity *K* = 0.25 show an acute reduction of *C*_*av*_ as well as of *R*_*av*_ (see results at *t* = 128 s in Figures 2A,B). Only the correlated multichannel noisy stimulation (CMNS) results in an increase of *C*_*av*_ and a small reduction of *R*_*av*_. The boxplots in Figures 2C,D show that these acute effects are representative for all 11 samples: compared to the control signal all stimulation protocols induce a statistically significant decrease of *C*_*av*_ and *R*_*av*_, except the CMNS which induces also a statistically significant decrease of *R*_*av*_, but a statistically significant increase of *C*_*av*_ (left-sided Wilcoxon rank sum test with α = 0.05). The corresponding *p*-values are given in Supplementary Table 1.

**Figure 3:**
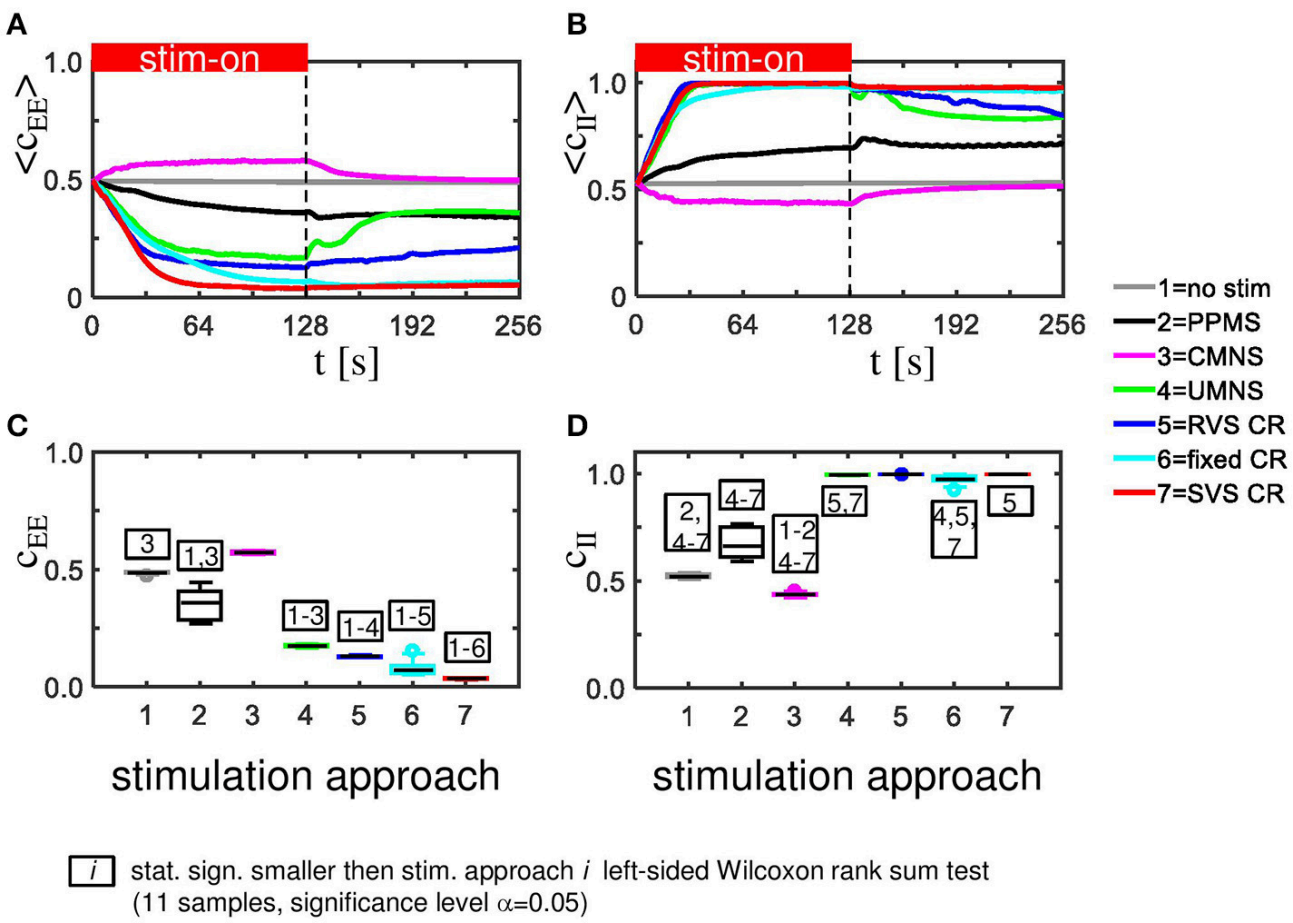
CMNS induces reversed changes in average excitatory, respectively, inhibitory synaptic weights (***c***_***EE***_, respectively ***c***_***II***_) compared to other stimulation protocols. All stimulation protocols, except the CMNS, show an acute and long-lasting decrease in excitatory synaptic weights **(A)** as well as an acute and long-lasting increase in inhibitory synaptic weights **(B)** of the same network as studied in Figures 2A,B. CMNS induces an increase of the average excitatory synaptic weights and a decrease of the average inhibitory synaptic weights. The red horizontal bar represents the stim-on period. Boxplots confirm the results for excitatory **(C)** as well as for inhibitory synaptic weights **(D)** (11 samples). *p*-values of the left-sided Wilcoxon rank sum test are given in Supplementary Table 1. All stimulations are applied at *K* = 0.25.

The CMNS-induced increase of *C*_*av*_ is the result of a statistically significant increase of the average excitatory synaptic weight *c*_*EE*_ in combination with a statistically significant decrease of the average inhibitory synaptic weight *c*_*II*_ (see Figures 3A,B for one set of initial network conditions and Figures 3C,D for all 11 samples). The other stimulation protocols show an opposite behavior: the combination of a statistically significant decrease of *c*_*EE*_ with a statistically significant increase of *c*_*II*_ (Figure 3) explains the statistically significant decreases of *C*_*av*_ (Figure 2C).

**Figure 4:**
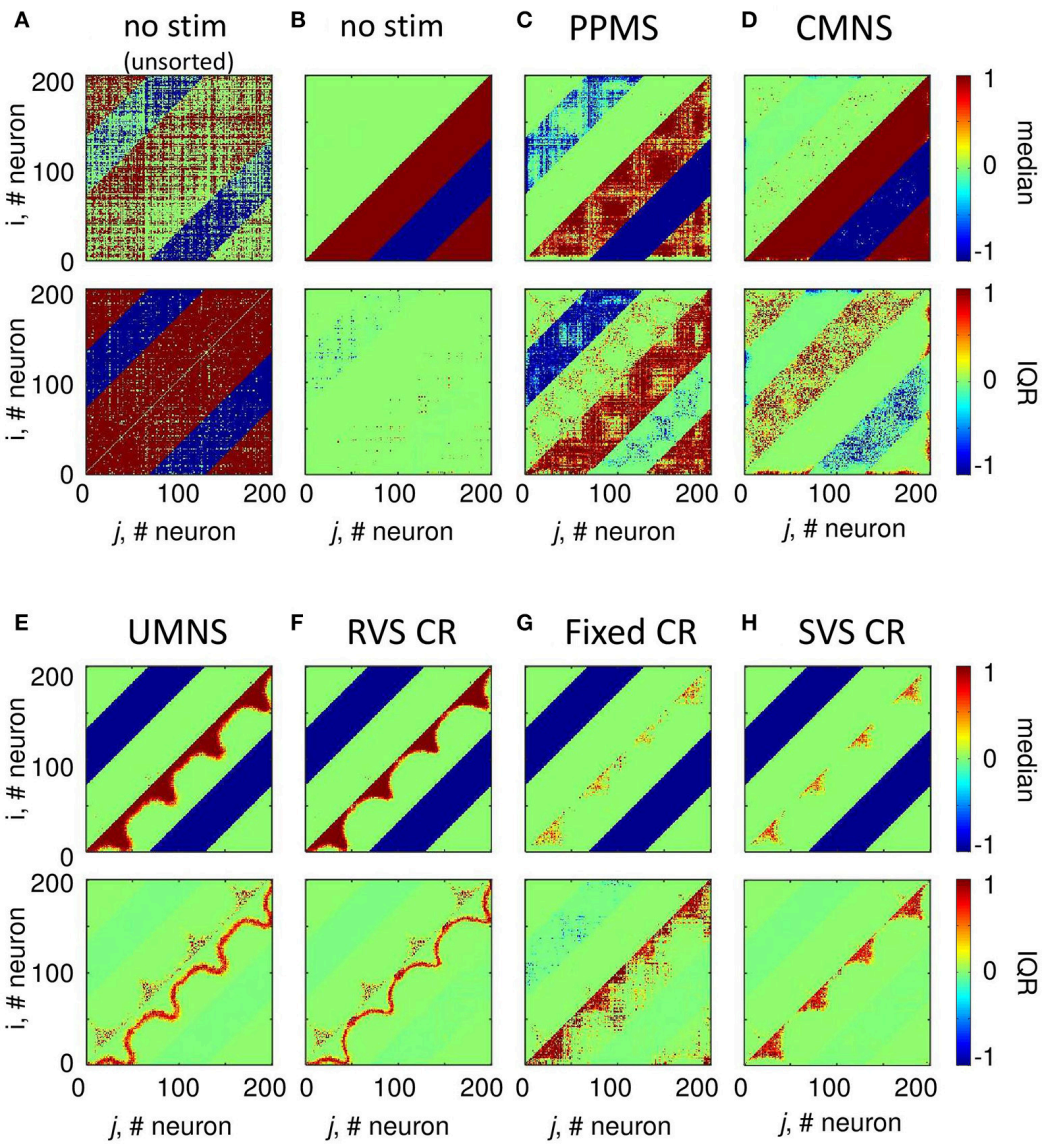
Small differences in stimulation protocols can result in very different connectivity patterns at the end of the stim-on period (*t* = 128 s). The median and IQR of the eleven unsorted connectivity matrices are shown in color code for the control signals (no-stim) **(A)**. The median and IQR of the eleven sorted connectivity matrices are shown for no-stim **(B)**, PPMS **(C)**, CMNS **(D)**, UMNS **(E)**, RVS CR **(F)**, fixed CR **(G)**, and SVS CR **(H)**. Negative values represent inhibitory synaptic weights, positive values relate to excitatory synaptic weights. All stimulation signals are applied with intensity *K* = 0.25. **(B–H)** are sorted matrices as described in the Methods section. The sorted matrices indicate whether or not a stimulation approach induces strong or weak bidirectional synapses and where the differences between samples occur.

According to the median of the unsorted connectivity matrices induced by the control protocol (no-stim) there are many strong excitatory (and inhibitory, respectively) synapses without a spatial pattern in relation to the stimulation sites at locations (25,25), (75,75), (125,125), and (175,175) (upper panel of Figure 4A). Note, the excitatory (respectively inhibitory) synapses have positive (respectively negative) values. The corresponding IQRs show that there are large differences between the synaptic weights induced by the different samples for almost all synapses (bottom panel of Figure 4A). By first sorting for each sample the *c*_*ij*_(*t* = 128 *s*) values such that the strongest synaptic weight between two neurons *i* and *j* is placed in the lower right triangle and the weaker synaptic weight between those two neurons *i* and *j* in the upper left triangle (see section Connectivity for more details) and then determining the median and IQR, it becomes clear that in general the control protocol results in only one strong synapse (|*c*_*ij*_| ≅ 1) between two neurons and in the reverse direction the synaptic weight is approximately zero (upper panel of Figure 4B). The corresponding IQR shows that the (sorted) synaptic strengths are rather independent of the actual sample (bottom panel of Figure 4B). So, the general pattern is that due to the control signals the synaptic weights between any two neurons are unidirectional (strong in one direction, weak in the reverse direction). This is similar for the synaptic weights at the beginning of the stim-on period (*t* = 0 s, result not shown).

Figure 4C shows that if all *N*_*s*_ = 4 stimulation sites are activated simultaneously and periodically (PPMS) more bidirectional strong inhibitory synapses exist at the end of the stim-on period and on the other hand some excitatory synapses are bidirectional and weak. Differences between the results induced by different samples are mainly found in the indices of the neurons which have bidirectional strong inhibitory synapses and in the indices of those neurons which have bidirectional weak excitatory synapses (Figure 4C).

In case all *N*_*s*_ = 4 stimulation sites are simultaneously stimulated in a noisy manner (CMNS), the median of the sorted connectivity matrices shows that besides the induction of some bidirectional strong excitatory synapses the median is similar to the median of the control signals (Figures 4B,D). However, the IQRs vary: compared to the no-stim condition (Figure 4B), different samples with CMNS will induce bidirectional strong excitatory synapses between different neuronal locations and also the induced bidirectional weak inhibitory synapses differ in locations (Figure 4D).

If the stimulation sites are not activated simultaneously but sequentially and still noisy (UMNS) then the number of unidirectional strong excitatory neurons decreases in such a way that they exist between neighboring neurons and between neurons nearby the stimulation sites, but not between more distant neurons (Figure 4E). Further, all inhibitory synapses are bidirectional and strong. Differences between samples are mainly found in the size of the region with strong excitatory synapses nearby the stimulation sites and between neighboring neurons and in the occurrence of bidirectional strong excitatory synapses nearby the stimulation sites (Figure 4E).

To some extent, RVS CR is similar to UMNS, but stimulation onsets are equidistantly spaced at 0, 4, 8, or 12 ms after onset of the ON-cycle instead of at random time instances as for UMNS. The median and IQR of the sorted connectivity matrices induced by RVS CR (Figure 4F) is similar to the results induced by UMNS. Small differences are obtained for the RVS CR compared to the UMNS: the unidirectional strong excitatory neurons exist in a smaller neighborhood of each neuron as well as of the stimulation sites. These small differences are in agreement with the decrease of excitatory synaptic weights in Figure 3C and with the smaller *C*_*av*_-values induced by RVS CR compared to those induced by UMNS in Figure 2C.

The fixed CR activates the stimulation sites sequentially at 0, 4, 8, and 12 ms after onset of the ON-cycle, while the sequence does not change during the stim-on periods. This repetition of sequence has several effects (compare Figures 4F,G). Most prominent is the fact that due to the fixed CR (Figure 4G) the neurons close to a stimulation site are not so strongly coupled as in case of the RVS CR (Figure 4F). Furthermore, in general, the neurons halfway between two stimulation sites are not strongly coupled any more with their direct neighbors. Due to the repetition of the sequence strong excitatory couplings between neurons surrounding two consecutively activated stimulation sites remain present. For the eleven different samples (each with another sequence), this leads to some large IQRs for couplings between more distant excitatory synapses. By changing now and then the sequence during the stim-on period (SVS CR), these large IQRs between more distant excitatory neurons disappear (Figure 4H).

**Figure 5:**
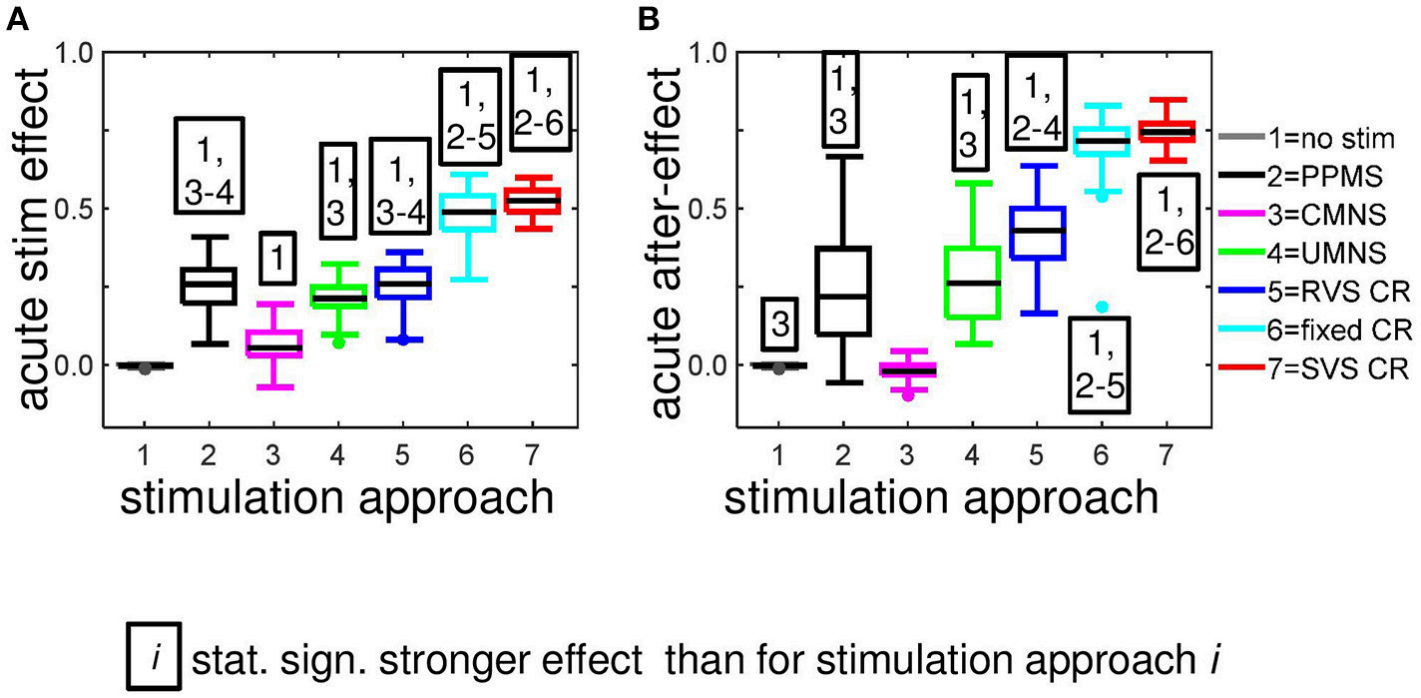
Most stimulation protocols induce acute stimulation- and acute after-effects. Boxplots show the distributions of the acute stimulation-effect **(A)** and the acute after-effect **(B)** induced in all subpopulations of the eleven networks by the different stimulation protocols (*n* = 44 samples). Acute stimulation effects are determined by Equation (16), acute after-effects by Equation (17). Statistically significantly stronger effects are determined by the right-sided Wilcoxon rank sum test with significance level α = 0.05 (see for *p*-values Supplementary Table 2). All stimulations are applied at *K* = 0.25.

In Figure 2D we have seen how the amount of synchronization of the complete network *R*_*av*_, changes under influence of the different stimulation approaches at stimulation intensity *K* = 0.25. On a mesoscopic level we investigate the desynchronizing effect in the four subpopulations containing *N*_*k*_ = 49 neurons near the stimulation sites. For each stimulation protocol Figure 5A illustrates at *K* = 0.25 the distribution of the *N*_*s*_ · *n* = 44 determined acute stimulation effects. All stimulation approaches induce a statistically significant acute stimulation effect whereby the weakest effect is induced by the CMNS (right-sided Wilcoxon rank sum test, α = 0.05, *n* = 44; see Supplementary Table 2 for *p*-values). The order of the stimulation protocols in having a stronger desynchronizing effect is clear for the macroscopic measure *R*_*av*_: only RVS CR and fixed CR have a similar effect on *R*_*av*_. On the mesoscopic level this order is slightly different: the PPMS turned out as good as RVS CR, while fixed CR caused a better desynchronization of the neuronal activity than RVS CR (compare Figures 2D, 5A; one-sided Wilcoxon rank sum test with α = 0.05; see Supplementary Table 1 respectively 2 for *p*-values corresponding by Figure 1D respectively Figure 5A).

**Figure 6:**
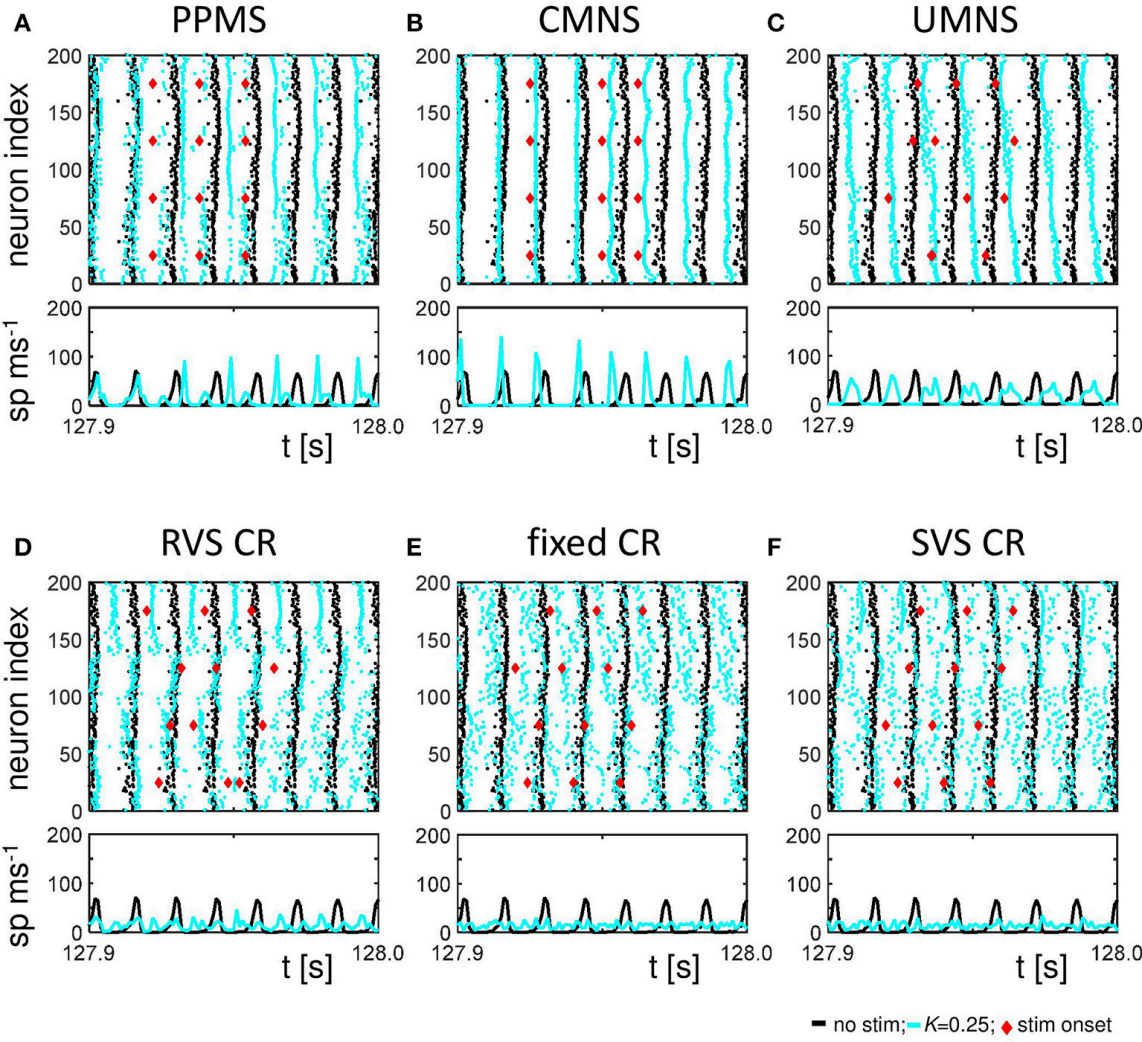
Raster plots and spike counts at the end of the stim-on period (*t* = 128 s) show that sequential stimulation causes a desynchronization, whereas simultaneous stimulation may even increase synchronization. **(A)** Raster plot and spike count (spikes/ms) of the last 100 ms of the 128 s stim-on period of the PPMS stimulation applied at *K* = 0.25 (blue dots) and the control signal (no-stim; *K* = 0.0, cyan dots). Each dot in the raster plot represents the spike time of the corresponding neuron. Each red diamond shows the stimulation onset of the corresponding stimulation site. Spike counts for the last 100 ms of the stim-on period show how many neurons fire within each time interval of 1 ms. **(B–F)** as **(A)** for CMNS **(B)**, UMNS **(C)**, RVS CR **(D)**, fixed CR **(E)**, and SVS CR **(F)**.

Figure 6 shows the raster plots and spike counts induced by the different stimulation protocols (*K* = 0.25) for the same samples as used in Figures 2A,B, 3A,B, 4 during the last 100 ms of the stim-on period. Activating the stimulation sites simultaneously does not cause a pronounced desynchronization (compare the results for no-stim with *K* = 0.25 in Figures 6A,B). A sequential random activation of the stimulation sites can broaden the synchronized spike-volley (Figure 6C). RVS-CR stimulation counteracts in-phase synchronization, typically by causing cluster states, where the network forms several phase-shifted (synchronized) subpopulations (see e.g., Figure 6D). A more pronounced overall desynchronization is achieved by means of the fixed CR stimulation and the SVS-CR stimulation (e.g., Figures 6E,F).

The spike counts suggest that activating the stimulation sites simultaneously can result in stronger synchronization of the activity of the whole network, while sequential stimulation can divide the network in several synchronized, but mutually phase-shifted subpopulations, which in turn causes a pronounced overall (i.e., close to uniform) desynchronization, as reflected by *R*_*av*_. Since each single stimulus synchronizes the nearby neurons, while desynchronizing the entire neuronal network, on a macroscopic scale the in-phase synchronization (and hence *R*_*av*_) may vanish, while the order parameters of the different subpopulations may still attain high values. Put otherwise, the acute stimulation effect will be weaker on the mesoscopic than on the macroscopic level (e.g., for UMNS and RVS CR). For the PPMS and the CMNS, which activate all stimulation sites simultaneously, the order parameter of each subpopulation represents the order parameter of the macroscopic network quite well and, hence, the acute stimulation effect is comparable on the mesoscopic and macroscopic level.

**Figure 7:**
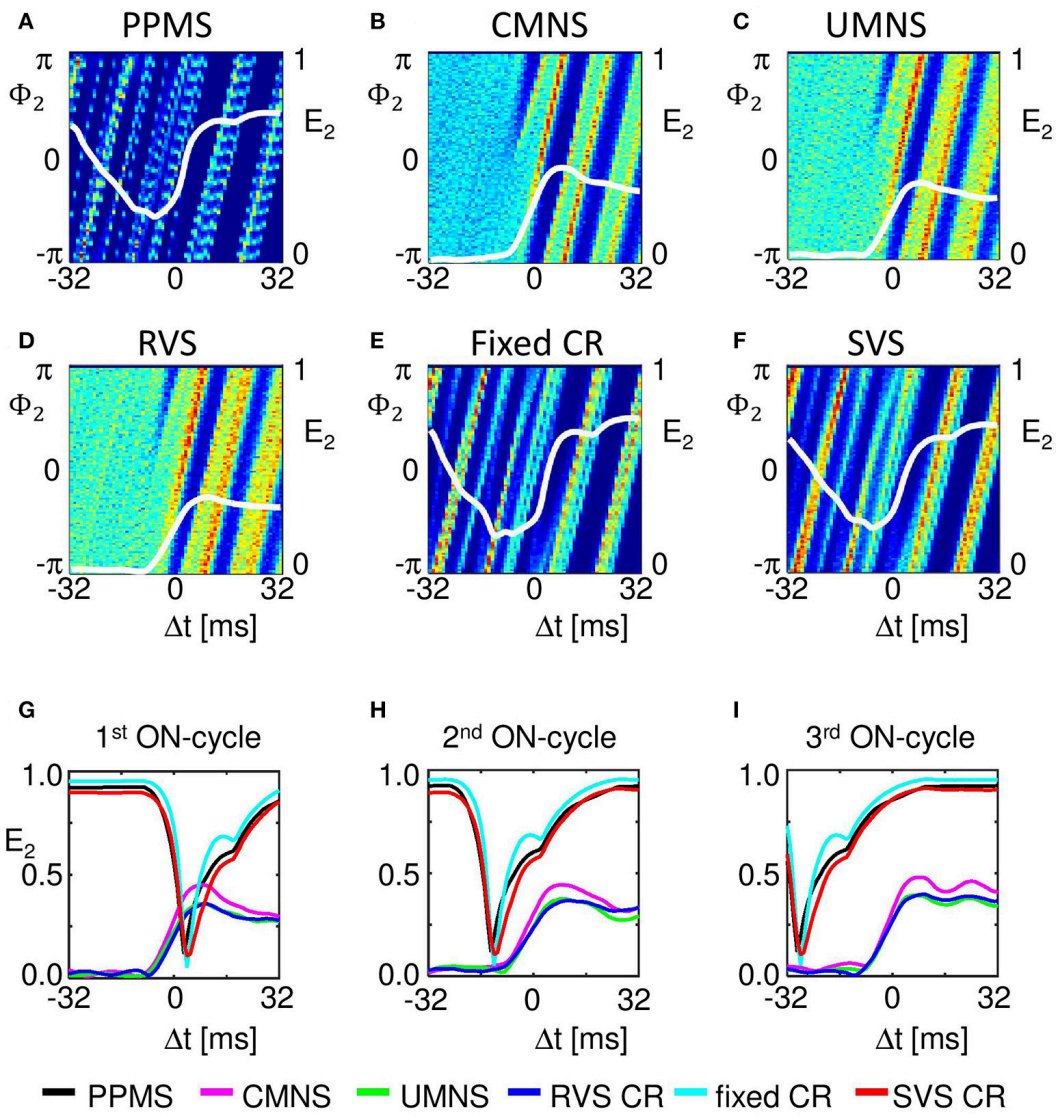
Periodic stimulus repetition results in an entrainment of the mean phase of the subpopulation by the stimulation signal, whereas varying inter-stimulus-intervals cause phase resets. Cross-trial distributions of the mean phase of subpopulation 2 (neurons 51–99) Φ_2_(Δ*t*), averaged across the 4,800 stimulation onsets of subpopulation 2 within a time window locked to the corresponding stimulus onsets for PPMS **(A)**, CMNS **(B)**, UMNS **(C)**, RVS CR **(D)**, fixed CR **(E)**, and SVS CR **(F)** are color coded with a minimum of zero (blue) and a maximum value (in red) of 404 **(A)**, 145 **(B)**, 117 **(C)**, 114 **(D)**, 238 **(E)**, and 192 **(F)**. The resetting index of subpopulation 2, *E*_2_, is shown by a white curve superimposed to each phase distribution diagram. The resetting index is also calculated for the distributions of Φ_2_(Δ*t*), averaged across the 1,600 stimulation onsets within the first **(G)**, the second **(H)**, and the third **(I)** of the three consecutive ON-cycles for the different stimulation protocols. *K* = 0.25 for all panels.

Our analysis continues on the mesoscopic level to study the mechanisms by which the different stimulation protocols influence the synchronization. For this we use the cross-trial analysis, which investigates the subpopulations' phases time-locked to the corresponding stimulus onset, averaged over all stimulus onsets of stimuli delivered to a particular subpopulation during the stim-on period. For subpopulation 2 (comprising neurons 51-99), Figures 7A–F show a clear difference between the stimulation protocols with periodic delivery pattern, PPMS, fixed CR and SVS CR, and those which have no strictly periodic stimulus delivery pattern (CMNS, UMNS, and RVS CR). The latter protocols cause phase resets of the stimulated subpopulations: Before stimulus onset (for Δ*t* < 0 m) the phase distributions are close to uniform, whereas after stimulus delivery a stereotypical restart of the subpopulation phase occurs, which is reflected by the emergence of a pronounced peak of the distribution (for Δ*t* > 0 ms in Figures 7B–D). In contrast, in case of the periodic stimulus patterns, PPMS, fixed CR and SVS CR, a pronounced peak of the phase distribution increasingly vanishes in the absence of stimulation (for Δ*t* < 0 m) and re-occurs after stimulus delivery (for Δ*t* > 0 ms) (Figures 7A,E,F). The corresponding resetting indices *E*_2_(Δ*t*) show that the non-periodic stimulation protocols induce a phase reset (Figures 7B–D): Following stimulus onset, the initially quite homogeneously distributed subpopulation phase turns into a unimodal phase distribution. In contrast, for the periodic stimulation protocols, the resetting indices display a completely different time course: Starting at a large value, they first decrease, then re-increase due to the first stimulus (at Δ*t* = 0 ms) and further increase due to the subsequent stimulus (Δ*t* ~ 16 ms) (Figures 7A,E,F). More precisely, at Δ*t* ~ 16 ms in only 2/3 of all stimulation onsets there is indeed a stimulation, since the third ON-cycle of each block of three consecutive ON-cycles is not directly followed by an ON-cycle but instead by two OFF-cycles. This implies that after three activations of the stimulation site within the subpopulation with an inter-stimulus-interval of 16 ms, the next inter-stimulus-interval is equal to 48 ms. Accordingly, by sorting the stimulation onsets according to their order in the blocks of three consecutive ON-cycles, reveals the effect of the different stimuli. The first of the three stimuli destroys most of the phase entrainment which is present during the OFF-cycles and then builds up the entrainment up to a lower level than the initial entrainment (Figure 7G). The second of the three stimuli further increases the extent of phase entrainment (Figure 7H), and the third stimulus finally increases the phase entrainment to the initial level (Figure 7I). The maximum values of the resetting indices of the periodic stimulation protocols increase within a block of three consecutive stimuli (i.e., ON-cycles) from one stimulus to the subsequent one, indicating a further increase of the phase entrainment between stimulated subpopulation and corresponding stimulus train (compare the maxima in Figures 7G–I). Accordingly, the effect of the three consecutive periodic stimuli on the phase builds up within each block. In contrast, each subsequent non-periodic stimulus causes a new phase reset (Figures 7G–I).

Sequential stimulation patterns (UMNS, RVS CR, fixed CR, SVS CR) cause a more pronounced reduction of synchrony and the mean synaptic weight on the network level (Figure 2) as well as on the subpopulation level (Figures 3, 5) compared to simultaneous stimulation patterns (PPMS, CMNS). On the level of the individual neurons the sequential stimulation patterns induce strong bidirectional inhibitory synapses and subpopulations with strong unidirectional excitatory coupling of the neurons near the stimulation sites, whereas the simultaneous stimulation protocols only induce some strong bidirectional inhibitory synapses and no spatial pattern of the strong unidirectional excitatory synaptic weights (compare Figures 4E–H with Figures 4C,D).

Another important aspect refers to the sequential arrangement of stimulation sequences. Repeating a sequence many times in a row (by fixed CR or SVS CR) causes a more pronounced reduction of *C*_*av*_ than random variations of the sequences (by UMNS or RVS CR) (Figure 2). Sufficient repetition of stimulation sequences results in a stronger reduction of *c*_*EE*_ (Figure 3) caused by the fact that less neurons are coupled within a subpopulation around each stimulation site together with a weaker synaptic strength for those who are coupled (Figure 4G,H compared with Figures 4E,F). Although on the network level no clear difference in the amount of desynchronization is observed (RVS CR and fixed CR induced similar *C*_*av*_ –values; Figure 2D), on the subpopulation level the repetitive sequences induce a stronger acute stimulation effect (Figure 5). This suggests that different mechanisms of action may cause different types of macroscopic desynchronization: desynchronization between subpopulations which themselves can still be highly synchronized as opposed to a more overall, uniform desynchronization, affecting the subpopulations as well. According to the raster plots in Figure 6, a more uniform desynchronization is typically observed as a result of repetitive sequence CR (fixed CR and SVS CR). Figure 7 shows that on the subpopulation level there is an entrainment between the stimulation signal and the subpopulation activity in the networks exposed to repetitive sequence CR. In contrast, phase resets are the salient mechanism of those CR variants without sequence repetition.

Intriguingly, all stimulation protocols except the CMNS protocol induce a decrease of *c*_*EE*_ and an increase in *c*_*II*_ compared to the no-stim protocol (Figure 3). In contrast, the CMNS protocol induces exactly the opposite (Figure 3), resulting in an increase of *C*_*av*_, in contrast to all other active stimulation protocols (Figure 2C). Since also the median of the sorted connectivity matrix induced by CMNS is similar to the one of the control (no-stim) signal and not for the other stimulation protocols (Figures 4B,D), the CMNS seems to be the best candidate for a sham stimulation protocol for sensory CR stimulation. While the stimulation shares some perceptual features with CR stimulation, the acute effects of CMNS on connectivity and desynchronization are minimal.

### Long-term effects

It is a key goal for non-invasive neuromodulation techniques to cause long-lasting, sustained effects that persist after cessation of stimulation. Sufficiently pronounced long-lasting effects may open up the possibility to deliver stimulation only regularly or occasionally e.g., for a few hours only, to maintain substantial relief. Therefore, we are particularly interested in the effects of the different stimulation protocols after withdrawal of stimulation. In this section, we study these long-term effects at the end of the stim-off period (*t* = 256 s) unless stated otherwise. Again, the stimulations during the stim-on period were applied at intensity *K* = 0.25.

**Figure 8:**
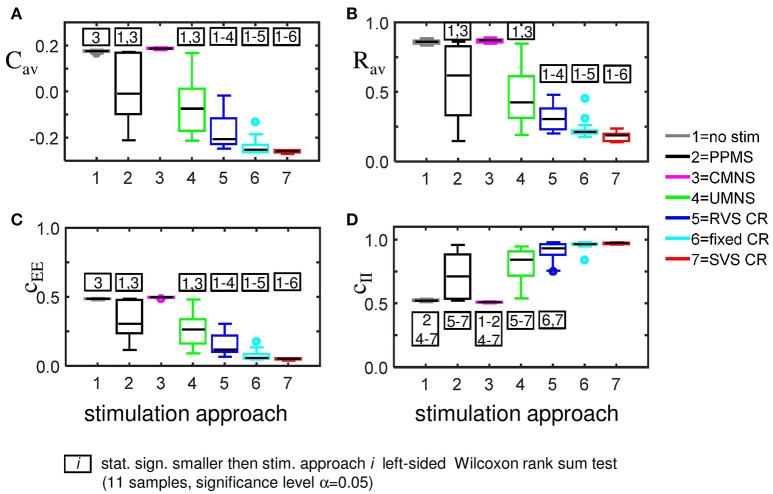
Long-lasting anti-kindling effects for different stimulation protocols. **(A)** Boxplots of *C*_*av*_ (*t* = 256 s) for different stimulation protocols show a statistically significant decrease compared to the control condition (no-stim) for the stimulation protocols with sequential activation of the four stimulation sites. In contrast, CMNS causes no decrease of *C*_*av*_. **(B)** Boxplots of *R*_*av*_ (*t* = 256 s) show a statistically significant desynchronization induced by the different stimulation protocols compared to the control signal (no-stim) except for the inert CMNS. All stimulation protocols, except CMNS, induce a long-lasting decrease in the average excitatory synaptic weight *c*_*EE*_ **(C)** and a long-lasting increase in the average inhibitory synaptic weight *c*_*II*_ **(D)**. Each boxplot represents the results of 11 samples. The horizontal line within the box represents the median, the length of the box the IQR (middle 50%), whereas the whiskers below and above the box indicate the first and last 25%. Outliers are defined as 1.5 times the length of the box below or above the box and are represented by open circles. *p*-values of the left-sided Wilcoxon rank sum test are given in Supplementary Table 3. *K* = 0.25 for all panels.

For the same initial network configuration, a stimulation epoch of duration *t* = 128 s can have different acute and long-term effects on the average synaptic weight *C*_*av*_, depending on the stimulation protocol selected (Figure 2A). To focus on the long-term effects, the distributions of the *C*_*av*_ (*t* = 256 s) at the end of the stim-off period are shown in Figure 8A for the different stimulation protocols. The SVS CR protocol induces the greatest reduction of *C*_*av*_ (*t* = 256 s), while the CMNS induces even a small increase. These long-term effects (Figure 8A) are qualitatively comparable with the acute effects (Figure 2A) and statistically significant. None of the stimulation protocols induces *C*_*av*_ effects statistically equivalent to the no-stim protocol. However, CMNS induces *R*_*av*_ (*t* = 256 s) values similar to the control signal (no-stim) (Figure 8B), and there are no statistically significant differences. In contrast, the SVS CR stimulation induces the smallest *R*_*av*_ values at *t* = 128 s (acute effect; Figure 2B) as well as at *t* = 256 s (long-term effect; Figure 8B). Therefore, the CMNS protocol turns out to be the best candidate for sham stimulation. The *p*-values obtained by the left-sided Wilcoxon rank sum tests for the long-term effects as shown in Figures 8A–D are given in Supplementary Table 3.

The strongest *C*_*av*_ is induced by the CMNS protocol (Figure 8A) and can be disentangled in the strongest average excitatory synaptic weight *c*_*EE*_ of the tested protocols (Figure 8C) in combination with the weakest inhibitory synaptic weight *c*_*II*_ of the tested protocols (Figure 8D). The weakest *C*_*av*_ as shown in Figure 8A is induced by the SVS CR stimulation protocol and is a combination of the weakest *c*_*EE*_ (Figure 8C) and the strongest *c*_*II*_ (Figure 8D) of the tested protocols. In general, a weaker *C*_*av*_ value obtained by a particular stimulation protocol compared to another protocol can be explained by a significantly reduced *c*_*EE*_ and increased *c*_*II*_ of the first stimulation protocol except for the comparison between fixed CR and SVS CR. These two protocols induce a similar *c*_*II*_ and thus only the stronger reduced *c*_*EE*_ induced by the SVS CR stimulation contributes to the smaller *C*_*av*_ value compared to the fixed CR stimulation (Figures 8A,C,D).

**Figure 9:**
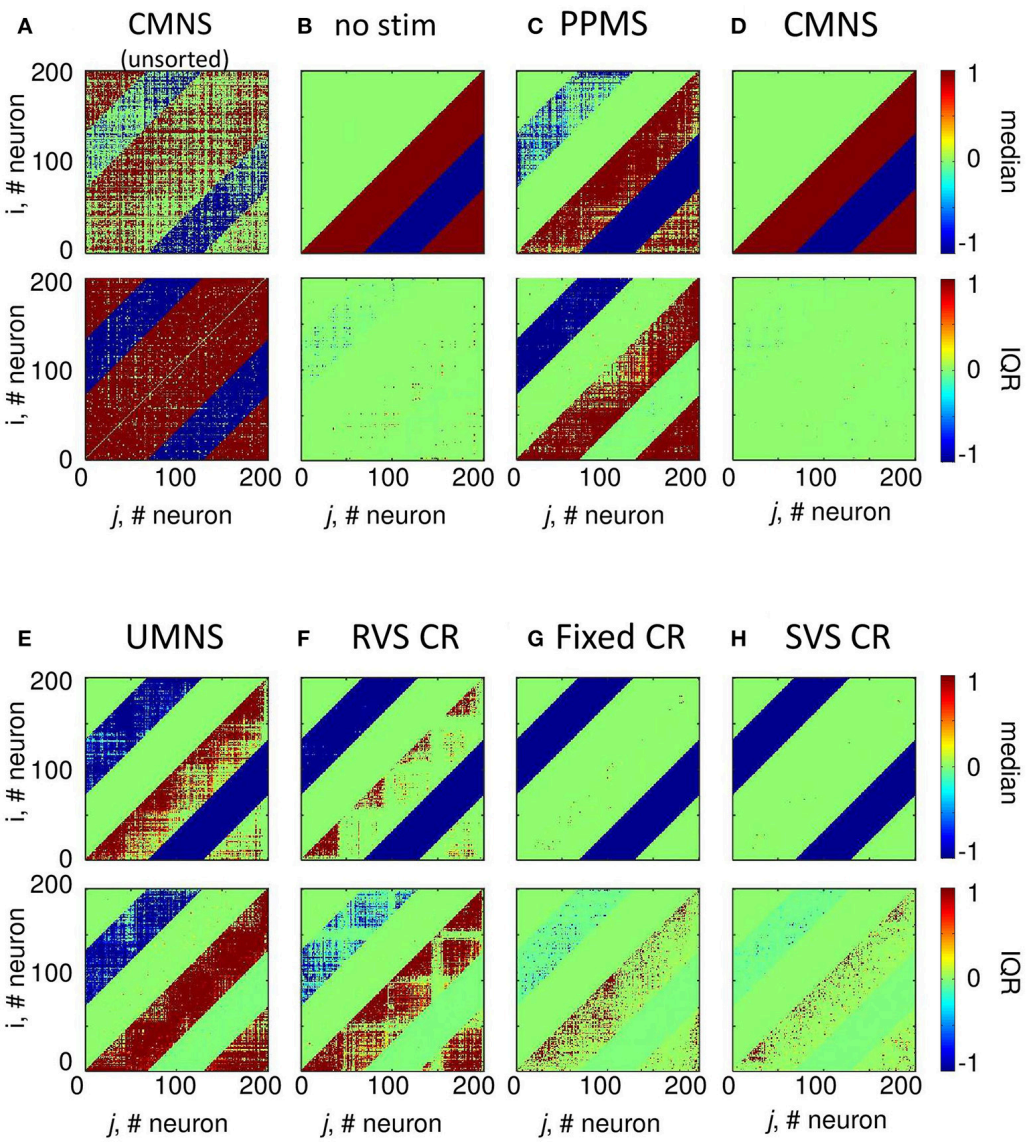
Sorted connectivity matrices at the end of the stim-off period (*t* = 256 s) differ from the control signal (no-stim) except for the CMNS protocol. **(A)** The median and IQR of the 11 unsorted connectivity matrices induced by the CMNS stimulation protocol are shown in color code. Negative values represent inhibitory synaptic weights, positive values excitatory ones. The median and IQR of the 11 sorted coupling matrices are color coded as in **(A)** for the control signal (no-stim) **(B)**, PPMS **(C)**, CMNS **(D)**, UMNS **(E)**, RVS CR **(F)**, fixed CR **(G)**, and SVS CR **(H)**. All stimulation signals are applied with intensity *K* = 0.25. **(B–H)** are sorted matrices as described in the Methods section.

Comparing the medians and IQRs of the unsorted connectivity matrices at *t* = 256 s (long-term effect) for CMNS (Figure 9A) with those for the no-stim protocol at *t* = 128 s (Figure 4A) show similar patterns. Note that the patterns do not change for the no-stim protocol at different times e.g., at *t* = 0 or 256 s (results not shown). At the end of the stim-off period the medians and IQRs of the sorted connectivity matrices of CMNS (Figure 9D) and of the no-stim protocol (Figure 9B) look also similar and are comparable to those of the no-stim protocol at the end of the stim-on period (Figure 4B): strong unidirectional synaptic connections, with only a very small difference between the sorted connectivity matrices of different samples. Compared to the acute effect induced by CMNS (Figure 4D) the network has lost its strong bidirectional excitatory synapses during the stim-off period (Figure 9D) for all samples.

In case the stimulation onsets of the simultaneous stimulation patterns are not random as for CMNS but periodic (PPMS), bidirectional strong inhibitory synapses exist at the end of the stim-off period and most excitatory synapses are unidirectional and strong, while some of them are bidirectional and weak (Figure 9C). Indices of the neurons which have bidirectional strong inhibitory synapses or bidirectional weak excitatory synapses can differ for other samples (Figure 9C).

For the CR protocols, the medians of the sorted connectivity matrices have lost their connectivity patterns of the short-range excitatory synapses during the stim-off period (compare Figures 9E–H with Figures 4E–H). Mainly the CR protocols without repetition (Figures 9E,F) show a loss of some bidirectional strong inhibitory synapses, which were formed during the stim-on period (Figures 4E,F). For the CR protocols the IQRs of the sorted connectivity matrices are different at the end of the stim-off period compared with those at the end of the stim-on period (compare the bottom panels of Figures 9E–H with those of Figures 4E–H): at the end of the stim-off period large IQR are not only found for the strongest synapse between neighboring neurons (diagonals) or nearby stimulation sites, but without a clear spatial pattern in the lower right triangle large IQRs can be found for the excitatory synapses and in the upper left triangle for the inhibitory synapses influenced mainly by UMNS or RVS CR (bottom panels of Figures 9E–H).

Although on the macroscopic neural network level there is no significant difference between the desynchronization induced by the CMNS and by the no-stim protocol (Figure 8B), on the subpopulation level, there is a statistically significant acute after-effect (Figure 5B): An increase in the amount of synchronization is induced by CMNS. All other stimulation protocols have a stronger desynchronizing effect than the no-stim protocol, whereby the acute after-effects of the RVS, fixed and SVS CR are increased compared to their acute stimulation effects (Figures 5A,B). These increases are statistically significant (see Supplementary Table 2 for the *p*-values of the right-sided Wilcoxon rank sum test with significance level α = 0.05).

Accordingly, we conclude that sequential activation of the stimulation sites also induces more pronounced long-term anti-kindling effects except for UMNS, which tends to induce better long-term anti-kindling effects than PPMS, but the improved medians are not statistically significant (network level see Figures 8A,B; subpopulation level see Figures 5B, 8C,D). At the end of the stim-off period the results induced by consecutively repeating each sequence many times (fixed CR and SVS CR) are still better than without repetition (UMNS and RVS CR) on the network level (Figures 8A,B), as well as on the subpopulation level (Figures 8C,D for the connectivity; Figure 5B for the desynchronizing effect).

Even at the end of the stim-off period the CMNS protocol results in the strongest *c*_*EE*_ and the weakest *c*_*II*_, and therefore gives rise to the largest *C*_*av*_ value of all stimulation protocols (Figures 8A,C,D). The long-term *R*_*av*_ shows no difference with the control signals (no-stim; Figure 8B), but it does show a synchronizing effect on the subpopulation level, which implies that within the subpopulations the amount of synchronization has increased compared to the beginning of the stim-on period (Figure 5B). Despite these small differences the median and IQR of the sorted connectivity matrices induced by the no-stim and CMNS protocols appear to be similar. From all the investigated stimulation protocols at *K* = 0.25 the CMNS protocol turns out to be the one which induces the most similar results as the control signal (no-stim), despite small but statistically significant differences.

### Robustness against stimulation intensity

**Figure 10:**
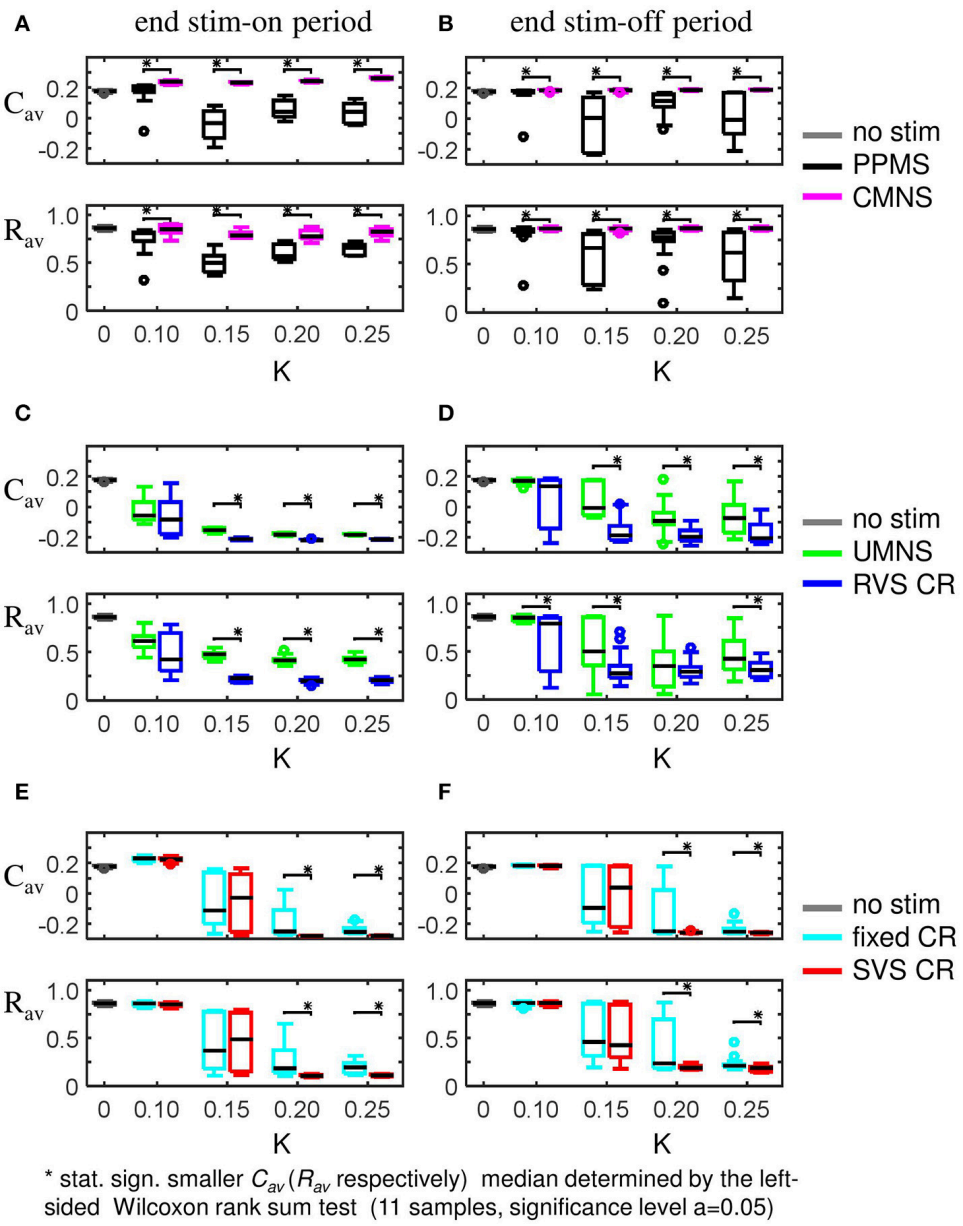
Boxplots of ***C***_*av*_ and ***R***_*av*_ show the effect of one difference between stimulus signal features. **(A,B)** PPMS and CMNS differ in the time between two consecutive stimulation onsets: PPMS has a constant period of 16 ms in consecutive ON-cycles, whereas CMNS has changing periods between stimulation onsets. The induced anti-kindling effects induced by PPMS are better than those induced by CMNS at the end of the stim-on period **(A)** as well as at the end of the stim-off period **(B)**. **(C,D)** For UMNS stimulation onsets are random and uncorrelated between different sites, whereas for RVS CR they are restricted to only four equidistant moments within the ON-cycle. RVS CR induces a stronger reduction of *C*_*av*_ and *R*_*av*_ than UMNS for most K-values at *t* = 128 s **(C)** as well as at *t* = 2 56 s **(D)**. **(E,F)** Fixed CR applies the same sequence during the stim-on period, whereas SVS CR randomly draws a new sequence after 100 consecutive repetitions of a sequence. For *K* = 0.10 and *K* = 0.15 there are no statistically significant differences in the distributions of *C*_*av*_ and *R*_*av*_ at *t* = 128 s **(E)** and *t* = 256 s **(F)**. However for *K* = 0.15 and *K* = 0.20 SVS CR reduces the medians and IQRs of *C*_*av*_ and *R*_*av*_ more than the fixed CR does. Each boxplots represents eleven samples. *p*-values of the left-sided Wilcoxon rank sum test are given in Supplementary Tables 1, 3, 5–7.

In this section, we investigate acute and long-term effects elicited by stimulation intensities weaker than *K* = 0.25. From the previous sections, we can conclude that a small difference in stimulation protocols might result in completely different acute as well as long-term effects. For instance, the simultaneous and noisy stimulation (CMNS) does not decrease *C*_*av*_ as the simultaneous and period stimulation (PPMS) does. By the same token, at weaker intensities (*K* = 0.10, 0.15, and 0.20) PPMS induces statistically significantly smaller values of *C*_*av*_ and *R*_*av*_ values than CMNS (Figures 10A,B). See Supplementary Tables 1, 3, 5–7 for the corresponding *p*-values of the left-sided Wilcoxon rank sum test with significance level α = 0.05. The basic difference in the stimulation protocol feature between UMNS and fixed CR is the same as between PPMS and CMNS, namely noise or periodic stimulation. By comparing the *p*-values for the UMNS and the fixed CR it follows that for *K* = 0.10 UMNS induces smaller *C*_*av*_ and *R*_*av*_ values than fixed CR (Supplementary Table 5), but for *K* = 0.25 it is just the other way around (Supplementary Tables 1,3). Since for the investigated *K*-range PPMS always induces smaller *C*_*av*_ and *R*_*av*_ values than the CMNS, we can only conclude that the effect of noise stimulation also depends on how the stimulation sites are activated: simultaneously or sequentially. In case of sequential stimulation it depends strongly on *K*.

Fixed CR and SVS CR differ in the number of different sequences applied to the network. For fixed CR one sequence is applied 4,800 time during the on-period, whereas for SVS CR each sequence is applied 100 times before the next sequence is applied. For weak stimulation intensities up to *K* = 0.15 there is no difference in the anti-kindling effects. In contrast, at higher intensities changing the sequence from time to time, as for SVS CR, decreases the *C*_*av*_ and *R*_*av*_ values even more (Figures 10E,F) than the fixed CR does.

**Figure 11:**
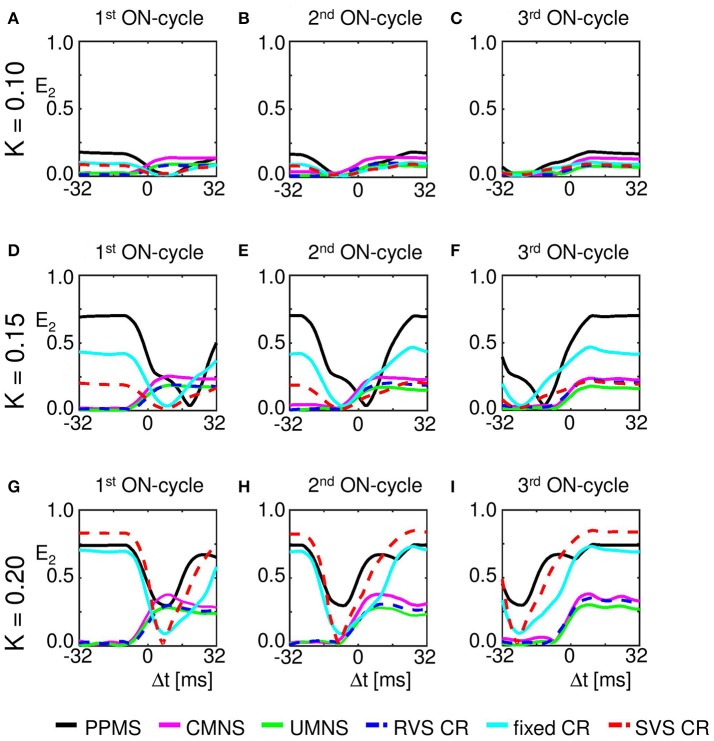
Maximum of the resetting index ***E***_2_ increases with stimulation intensity ***K***. **(A)** The resetting index within a window around the stimulus onset of the first ON-cycles of the three consecutive ON-cycles (in total 1,600 first ON-cycles) for different stimulation protocols at *K* = 0.10. **(B)** as **(A)** for the second of the three consecutive ON-cycles. **(C)** as **(A)** for the third of the three consecutive ON-cycles. **(D)** as **(A)** for *K* = 0.15. **(E)** as **(B)** for *K* = 0.15. **(F)** as **(C)** for *K* = 0.15. **(G)** as **(A)** for *K* = 0.20. **(H)** as **(B)** for *K* = 0.20. **(I)** as **(C)** for *K* = 0.20. The RVS and SVS CR results are shown by a dashed curve to improve visibility of the UMNS and fixed CR results.

Cross-trial analysis shows that already for *K* = 0.10 a weak phase reset is observed for CMNS, UMNS, and RVS CR and a weak entrainment followed by a stimulus-locked disruption of the weak phase entrainment for PPMS, fixed CR, and SVS CR (Figure 11). Increasing the intensity also increases the amount of entrainment as well as the strength of the phase reset (e.g., Figures 11A,D,G). During the first ON-cycle, PPMS, fixed CR as well as SVS CR stimulation destroy the entrainment of the subpopulation phase-dynamics with the stimulation signal in the entire investigated *K*-range. During the second ON-cycle, this entrainment is partly recovered by the stimulation except for stimulations at *K* = 0.15. In the latter case the entrainment is destroyed even further. During the third ON-cycle entrainment is restored in the entire *K*-range (*K* ∈ {0.10;0.15;0.20;0.25}), where the amount of entrainment increases with increasing *K*. The phase rest seems to increase slightly from the first to the second and, finally, to the third ON-cycle, but clearly increases with increasing *K* (*K* ∈ {0.10;0.15;0.20;0.25}).

## Discussion

We studied acute and long-lasting effects of six different stimulation protocols and compared the observed effects on a plastic neural network with a no-stimulation control condition. While sharing the same average rate of stimuli per channel, the tested stimulation protocols differ with respect to their amount of periodicity as opposed to randomness, both between ON-cycles and between stimulation sites. One of the tested stimulation protocols, CMNS, turned out not to induce desynchronization. In fact, comparing CMNS with the no-stimulation control condition showed that CMNS is nearly inert. More precisely, during stimulation (Figure 2), CMNS caused a significant increase of the strength of the mean synaptic connectivity by 49% compared to the no-stimulation control condition. Intriguingly, during CMNS the overall synchrony nevertheless was 8% smaller than for the no-stimulation control. Remarkably, there was hardly any long-term post-stimulation effect of CMNS compared to the no-stimulation control condition (Figure 8): While the mean synaptic weight increased significantly, but only slightly by 7%, the spontaneous (i.e., stimulation-free) synchronization did not significantly differ between the CMNS and the no-stimulation condition. Whether a slight, but significant increase of the mean synaptic weight might be relevant in a non-spontaneous context, where the network is, e.g., subjected to other types of stimuli, remains to be tested. Ultimately, clinical studies will provide the necessary tests.

In this study, we have shown that stimulation protocols, that differ by just one, putatively minor feature, may cause massively different anti-kindling effects, robustly over a range of stimulation intensities as well as for different samples at the end of the stim-on period and also at the end of the stim-off period (see e.g., Figure 10). Stimulating simultaneously or sequentially has a big influence on the synchronization and connectivity of the network at all levels (e.g., compare CMNS vs. UMNS in Figures 2C,D, 3C,D, 4D,E, 5, 6B,C, 8, 9D,E, 10). Another influential feature of some stimulation protocols is repetition (compare e.g., PPMS vs. CMNS or RVS vs. fixed CR), which goes along with a different dynamical stimulation mechanism: entrainment caused by repetition vs. phase reset otherwise (Figures 7, 10). In this study, we restricted ourselves to cycle durations of 16 ms, which is slightly above the intrinsic firing period of the individual neurons (14 ms). For a more pronounced mismatch of ON-cycle duration and firing periods, stimulus-locked entrainment of subpopulations may probably become more difficult if not completely impossible. Another drawback of the SVS and fixed CR is that they have to be applied at slightly stronger intensities than UMNS and RVS to be effective. However, SVS and fixed CR may, in principle, induce more pronounced long-term anti-kindling effects than UMNS and RVS CR (see e.g., Figures 8A,B).

Compared to all stimulation protocols tested in this study, the anti-kindling effects induced by the CMNS protocol are most similar to the control (i.e., no-stim) protocol (Figures 2C,D, 3C,D, 4D,E, 5, 6B,C, 8, 9D,E, 10), although the similarity is not always statistically significant. The CMNS protocol is also the only protocol which induced opposite effects on the connectivity, both on the network as well as subpopulation level, compared to all other active protocols (Figures 3, 8). Although the implementation of the stimulation in the model used here is for sensory stimulation, we expect similar results for electrical stimulation. For comparison between electrical and sensory stimulation effects and their qualitative similarities in our model, see Popovych and Tass (2012).

In addition, we also performed simulations which extended the finished trials with the CMNS protocol (*K* = 0.20) by adding a second, additional 128 s lasting stim-on period at *t* = 256 s with the RVS CR protocol as well as with the SVS CR protocol, each at *K* = 0.20. Anti-kindling effects induced at *t* = 384 s (i.e., at the end of the second stim-on period) as well as at *t* = 512 s (the end of the second stim-off period) were statistically significantly similar with the anti-kindling effects at *t* = 128 s (the end of the first and only stim-on period) and *t* = 256 s (the end of the first and only stim-off period) for the RVS and SVS CR only protocols applied during only one stim-on period as shown in Figures 10C–F (data not shown). Accordingly, from a computational standpoint there is no reason to assume that application of CMNS sham stimulation might render subsequent delivery of RVS or SVS CR stimulation ineffective.

Apart from the stimulation-related aspects, our findings are relevant with respect to the assessment of changes of synchronization and synaptic connectivity patterns and their mutual interrelation. Although the macroscopic measures *C*_*av*_ and *R*_*av*_ were often strongly correlated, an increase in *C*_*av*_ did not necessarily imply an increase in *R*_*av*_. For example, in Supplementary Table 4, we show that for the CMNS protocol applied at *K* = 0.25 during the stim-off period a statistically significant decrease in *C*_*av*_ is combined with a statistically significant increase in *R*_*av*_. Accordingly, it is not sufficient to exclusively monitor the connectivity in our model. Rather, we also have to determine the amount of synchronization. By a similar token, comparable amounts of macroscopic synchronization/desynchronization, as assessed by the order parameter, may differ on the mesoscopic, i.e., subpopulation level (see e.g., Figure 7). Accordingly, there is not just one type of (e.g., uniform) desynchronization. In summary, the analysis of both synchrony and synaptic connectivity on macroscopic as well as mesoscopic network levels may further the development of both active and inactive (sham) stimulation protocols.

In the field of drug development, a placebo treatment is realized by delivering a substance with no active therapeutic effect (Friedman et al., 2015). Accordingly, placebo effects can, for instance, be assessed with a parallel group design by comparing a placebo group with a natural history (i.e., no-treatment) group (Wager and Atlas, 2015). According to our computational results, CMNS is a promising candidate for a sham (i.e., inactive) stimulation protocol in the field of desynchronizing multi-channel stimulation. In the present study, we did not aim at developing a biophysical, microscopic model. Rather, we used a minimal model, equipped with robust spontaneous multistable dynamics, comprising synchronized and desynchronized states, that served as testbed for different stimulation protocols to generate first and experimentally testable hypotheses. By a similar token, several previous computational studies in the field of CR stimulation were carried out in minimal models and led to a number of clinically significant predictions, e.g., concerning cumulative effects and stimulation intensity (Hauptmann and Tass, 2009; Lysyansky et al., 2011), that were verified in pre-clinical and clinical studies (Tass et al., 2012b; Adamchic et al., 2014; Wang et al., 2016). In the same manner, the present computational study yields the testable hypothesis that CMNS might serve as sham stimulation protocol. Accordingly, the effects of CMNS should be tested in pre-clinical and, in particular, in clinical studies in order to disentangle CMNS effects from placebo effects in the best possible way. For instance, in a phase 1 (first in man) study feasibility and tolerability could be tested. In addition, in a phase 2 study an assessment of effects should be performed, possibly in comparison to a no-stimulation control and/or an active control group. Obviously, this adds to the complexity of the clinical development of neuromodulation treatments.

Furthermore, CMNS may be useful in clinical studies focusing on revealing predictive EEG markers for optimizing stimulation parameters of desynchronizing neuromodulation interventions, see Adamchic et al (2017). CMNS does neither cause substantial acute nor long-lasting effects. Nevertheless, CMNS might have unspecific EEG effects, e.g., on brain rhythms and/or brain areas less important to the disease related network dynamics. In this way, CMNS might help to separate EEG responses related to core stimulation effects from concomitant EEG responses. In addition, CMNS may also be helpful in pre-clinical studies to elucidate mechanisms by which stimulation protocols cause a desynchronization. Furthermore, CMNS might also help to reveal mechanisms that might be related to, but go beyond desynchronization, such as therapeutic rewiring (Tass and Majtanik, 2006) or neuroprotective effects (Musacchio et al., 2017).

Wherever appropriate and possible, sham procedures and double-blind protocols should be developed to scrutinize specific effects of neuromodulation interventions by adequate clinical trials and rule out placebo effects. However, placebo effects should not just be considered as a nuisance, requiring cumbersome clinical study protocols. Rather, the mechanisms underlying different placebo effects (Benedetti et al., 2011) could actually be specifically exploited to better neuromodulation techniques. For instance, similar to conditioned immunomodulation (Metal'nikov and Chorine, 1926; Ader and Cohen, 1975; Ader, 2003), one could condition specific desynchronization stimulation delivered invasively and causing long-lasting effects with unspecific non-invasive, sensory stimuli (Tass, 2011).

For the clinical implementation of multichannel sham stimulation protocols, one should take into account possible side effects related to the technical generation of the stimuli. For instance, in the field of transcranial magnetic stimulation (TMS) the current gold standard for sham TMS appears to be the use of a shielded coil, generating characteristic stimulus-related auditory stimuli, but no magnetic brain stimuli, together with surface electrodes for skin stimulation, mimicking magnetic skin stimulation (Duecker and Sack, 2015). Intriguingly, sham TMS may have specific side effects (Duecker and Sack, 2013, 2015). Obviously, TMS is not just a purely magnetic stimulation modality, but may constitute a compound stimulation approach which may cause specific effects caused by stimuli of different modality (Duecker and Sack, 2013, 2015). For the clinical development of multichannel sham stimulation protocols such aspects might be relevant, for instance, when vibrotactile mulitchannel stimulation causes auditory and possibly other sensory side effects.

This computational study is a first step for the development of a sham stimulation protocol for multichannel desynchronizing stimulation techniques. For comparison, for the development of CR stimulation, in computational studies predominantly minimal models were used (Tass, 2003a,b; Tass and Majtanik, 2006; Hauptmann and Tass, 2007, 2009; Lysyansky et al., 2011; Popovych and Tass, 2012; Zeitler and Tass, 2015, 2016), as opposed to biophysically realistic models (Ebert et al., 2014). These computational studies revealed non-trivial predictions, e.g., concerning the emergence of long-lasting, sustained (Tass and Majtanik, 2006) as well as cumulative (Hauptmann and Tass, 2009) effects and concerning the amplitude of the stimulation amplitude (Lysyansky et al., 2011). These predictions were verified in pre-clinical (Tass et al., 2012b; Wang et al., 2016) and clinical studies (Tass et al., 2012a; Adamchic et al., 2014; Syrkin-Nikolau et al., 2018). The computational predictions were used as hypotheses for the design of the corresponding study protocols. Analogously, the development of sham stimulation protocols requires a combined effort, comprising computational, pre-clinical, and clinical studies. The effect of a sham stimulation protocol may depend on the type of the stimulated neurons, the target area of the stimulation and the mechanism of the stimulation. Accordingly, future studies should also use other neuronal network models, e.g., network of FitzHugh-Rinzel bursting neurons (Rinzel, 1987; Izhikevich, 2001). By the same token, sham stimulation should ideally be inactive also in the presence of additional features and mechanisms, such as synaptic noise (Destexhe et al., 2003), propagation delays (Madadi Asl et al., 2017) as well for different stimulation mechanisms, e.g., excitatory vs. inhibitory stimulation (Popovych and Tass, 2012). Hence, future computational studies should take into account these refinements, too.

## Author contributions

PT came up with the initial ideas for this work; MZ designed and performed the simulations and analyzed the data; MZ and PT discussed findings and interpretations, and wrote the manuscript.

### Conflict of interest statement

The authors declare that the research was conducted in the absence of any commercial or financial relationships that could be construed as a potential conflict of interest.
