# Supplementary material for: Computationally Developed Sham Stimulation Protocol for Multichannel Desynchronizing Stimulation
Source: Front Physiol. 2018 May 8;9:512. doi: 10.3389/fphys.2018.00512 (PMC5952302; doi:10.3389/fphys.2018.00512)
Supplement: Supplementary file 1 [file Data_Sheet_1.docx]

Supplementary Material

Stimulation with similar spatio-temporal characteristics may have different acute and long-lasting effects

Magteld Zeitler, Peter A. Tass^*^

*** Correspondence:** Corresponding Author: Peter A. Tass, [ptass@stanford.edu](mailto:ptass@stanford.edu)

# Supplementary Tables

**Supplementary Table 1**: *p*-values belonging to **Figures 2C,D,** **3C,D, 10** obtained by the left-sided Wilcoxon rank sum test for different stimulation approaches with stimulation intensity *K* = 0.25 at the end of the stim-on period. *p*-values of smaller values which are statistically significant are highlighted in bold (significance level α = 0.05).

| C_av_ (128 s) | no stim | PPMS | CMNS | UMNS | RVS CR | fixed CR | SVS CR |
| --- | --- | --- | --- | --- | --- | --- | --- |
| no stim | 0.53 | 1.00 | **0.00** | 1.00 | 1.00 | 1.00 | 1.00 |
| PPMS | **0.00** | 0.53 | **0.00** | 1.00 | 1.00 | 1.00 | 1.00 |
| CMNS | 1.00 | 1.00 | 0.53 | 1.00 | 1.00 | 1.00 | 1.00 |
| UMNS | **0.00** | **0.00** | **0.00** | 0.53 | 1.00 | 1.00 | 1.00 |
| RVS CR | **0.00** | **0.00** | **0.00** | **0.00** | 0.53 | 1.00 | 1.00 |
| fixed CR | **0.00** | **0.00** | **0.00** | **0.00** | **0.00** | 0.53 | 1.00 |
| SVS CR | **0.00** | **0.00** | **0.00** | **0.00** | **0.00** | **0.00** | 0.53 |

| R_av_ (128 s) | no stim | PPMS | CMNS | UMNS | RVS CR | fixed CR | SVS CR |
| --- | --- | --- | --- | --- | --- | --- | --- |
| no stim | 0.53 | 1.00 | 0.99 | 1.00 | 1.00 | 1.00 | 1.00 |
| PPMS | **0.00** | 0.53 | **0.00** | 1.00 | 1.00 | 1.00 | 1.00 |
| CMNS | **0.01** | 1.00 | 0.53 | 1.00 | 1.00 | 1.00 | 1.00 |
| UMNS | **0.00** | **0.00** | **0.00** | 0.53 | 1.00 | 1.00 | 1.00 |
| RVS CR | **0.00** | **0.00** | **0.00** | **0.00** | 0.53 | 0.72 | 1.00 |
| fixed CR | **0.00** | **0.00** | **0.00** | **0.00** | 0.30 | 0.53 | 1.00 |
| SVS CR | **0.00** | **0.00** | **0.00** | **0.00** | **0.00** | **0.00** | 0.53 |

| <c_EE_(128 s)> | no stim | PPMS | CMNS | UMNS | RVS CR | fixed CR | SVS CR |
| --- | --- | --- | --- | --- | --- | --- | --- |
| no stim | 0.53 | 1.00 | **0.00** | 1.00 | 1.00 | 1.00 | 1.00 |
| PPMS | **0.00** | 0.53 | **0.00** | 1.00 | 1.00 | 1.00 | 1.00 |
| CMNS | 1.00 | 1.00 | 0.53 | 1.00 | 1.00 | 1.00 | 1.00 |
| UMNS | **0.00** | **0.00** | **0.00** | 0.53 | 1.00 | 1.00 | 1.00 |
| RVS CR | **0.00** | **0.00** | **0.00** | **0.00** | 0.53 | 1.00 | 1.00 |
| fixed CR | **0.00** | **0.00** | **0.00** | **0.00** | **0.00** | 0.53 | 1.00 |
| SVS CR | **0.00** | **0.00** | **0.00** | **0.00** | **0.00** | **0.00** | 0.53 |

| <c_II_(128 s)> | no stim | PPMS | CMNS | UMNS | RVS CR | fixed CR | SVS CR |
| --- | --- | --- | --- | --- | --- | --- | --- |
| no stim | 0.53 | **0.00** | 1.00 | **0.00** | **0.00** | **0.00** | **0.00** |
| PPMS | 1.00 | 0.53 | 1.00 | **0.00** | **0.00** | **0.00** | **0.00** |
| CMNS | **0.00** | **0.00** | 0.53 | **0.00** | **0.00** | **0.00** | **0.00** |
| UMNS | 1.00 | 1.00 | 1.00 | 0.53 | **0.00** | 1.00 | **0.01** |
| RVS CR | 1.00 | 1.00 | 1.00 | 1.00 | 0.53 | 1.00 | 1.00 |
| fixed CR | 1.00 | 1.00 | 1.00 | **0.00** | **0.00** | 0.53 | **0.00** |
| SVS CR | 1.00 | 1.00 | 1.00 | 1.00 | **0.01** | 1.00 | 0.53 |

**Supplementary Table 2**: *p*-values belonging to the acute stimulation effects and the acute after-effects for different stimulation approaches at *K* = 0.25 as shown in **Figure 5**. *p*-values are obtained by the *right*-sided Wilcoxon rank (*n* = 44). *p*-values of greater values which are statistically significant are highlighted in bold (significance level α = 0.05).

| Acute stim effects | no stim | PPMS | CMNS | UMNS | RVS CR | fixed CR | SVS CR |
| --- | --- | --- | --- | --- | --- | --- | --- |
| no stim | 0.50 | 1.00 | 1.00 | 1.00 | 1.00 | 1.00 | 1.00 |
| PPMS | **0.00** | 0.50 | **0.00** | **0.01** | 0.62 | 1.00 | 1.00 |
| CMNS | **0.00** | 1.00 | 0.50 | 1.00 | 1.00 | 1.00 | 1.00 |
| UMNS | **0.00** | 0.99 | **0.00** | 0.50 | 1.00 | 1.00 | 1.00 |
| RVS CR | **0.00** | 0.38 | **0.00** | **0.00** | 0.50 | 1.00 | 1.00 |
| fixed CR | **0.00** | **0.00** | **0.00** | **0.00** | **0.00** | 0.50 | 0.99 |
| SVS CR | **0.00** | **0.00** | **0.00** | **0.00** | **0.00** | **0.01** | 0.50 |

| Acute after-effects | no stim | PPMS | CMNS | UMNS | RVS CR | fixed CR | SVS CR |
| --- | --- | --- | --- | --- | --- | --- | --- |
| no stim | 0.50 | 1.00 | **0.00** | 1.00 | 1.00 | 1.00 | 1.00 |
| PPMS | **0.00** | 0.50 | **0.00** | 0.80 | 1.00 | 1.00 | 1.00 |
| CMNS | 1.00 | 1.00 | 0.50 | 1.00 | 1.00 | 1.00 | 1.00 |
| UMNS | **0.00** | 0.20 | **0.00** | 0.50 | 1.00 | 1.00 | 1.00 |
| RVS CR | **0.00** | **0.00** | **0.00** | **0.00** | 0.50 | 1.00 | 1.00 |
| fixed CR | **0.00** | **0.00** | **0.00** | **0.00** | **0.00** | 0.50 | 0.99 |
| SVS CR | **0.00** | **0.00** | **0.00** | **0.00** | **0.00** | **0.01** | 0.50 |

| $\frac{Acute stim effects}{Acute after-effects\backslash}$ | x=  no stim | x=  PPMS | x=  CMNS | x=  UMNS | x=  RVS CR | x=  fixed CR | x=  SVS CR |
| --- | --- | --- | --- | --- | --- | --- | --- |
| x | 0.54 | 0.78 | 1.00 | 0.12 | **0.00** | **0.00** | **0.00** |

| $\frac{Acute after-effects}{Acute stim effects\backslash}$ | x=  no stim | x=  PPMS | x=  CMNS | x=  UMNS | x=  RVS CR | x=  fixed CR | x=  SVS CR |
| --- | --- | --- | --- | --- | --- | --- | --- |
| x | 0.47 | 0.23 | **0.00** | 0.88 | 1.00 | 1.00 | 1.00 |

**Supplementary Table 3**: *p*-values belonging to **Figures 8, 10** obtained by the left-sided Wilcoxon rank sum test for different stimulation approaches with stimulation intensity *K* = 0.25 at the end of the stim-off period. *p*-values of smaller values which are statistically significant are highlighted in bold (significance level α = 0.05).

| C_av_ (256 s) | no stim | PPMS | CMNS | UMNS | RVS CR | fixed CR | SVS CR |
| --- | --- | --- | --- | --- | --- | --- | --- |
| no stim | 0.53 | 1.00 | **0.00** | 1.00 | 1.00 | 1.00 | 1.00 |
| PPMS | **0.00** | 0.53 | **0.00** | 0.91 | 1.00 | 1.00 | 1.00 |
| CMNS | 1.00 | 1.00 | 0.53 | 1.00 | 1.00 | 1.00 | 1.00 |
| UMNS | **0.00** | 0.10 | **0.00** | 0.53 | 0.99 | 1.00 | 1.00 |
| RVS CR | **0.00** | **0.00** | **0.00** | **0.01** | 0.53 | 1.00 | 1.00 |
| fixed CR | **0.00** | **0.00** | **0.00** | **0.00** | **0.00** | 0.53 | 0.98 |
| SVS CR | **0.00** | **0.00** | **0.00** | **0.00** | **0.00** | **0.02** | 0.53 |

| R_av_ (256 s) | no stim | PPMS | CMNS | UMNS | RVS CR | fixed CR | SVS CR |
| --- | --- | --- | --- | --- | --- | --- | --- |
| no stim | 0.53 | 1.00 | 0.08 | 1.00 | 1.00 | 1.00 | 1.00 |
| PPMS | **0.00** | 0.53 | **0.00** | 0.91 | 0.99 | 1.00 | 1.00 |
| CMNS | 0.93 | 1.00 | 0.53 | 1.00 | 1.00 | 1.00 | 1.00 |
| UMNS | **0.00** | 0.10 | **0.00** | 0.53 | 0.98 | 1.00 | 1.00 |
| RVS CR | **0.00** | **0.01** | **0.00** | **0.02** | 0.53 | 0.99 | 1.00 |
| fixed CR | **0.00** | **0.00** | **0.00** | **0.00** | **0.01** | 0.53 | 0.99 |
| SVS CR | **0.00** | **0.00** | **0.00** | **0.00** | **0.00** | **0.01** | 0.53 |

| <c_EE_(256 s)> | no stim | PPMS | CMNS | UMNS | RVS CR | fixed CR | SVS CR |
| --- | --- | --- | --- | --- | --- | --- | --- |
| no stim | 0.53 | 1.00 | **0.00** | 1.00 | 1.00 | 1.00 | 1.00 |
| PPMS | **0.00** | 0.53 | **0.00** | 0.88 | 1.00 | 1.00 | 1.00 |
| CMNS | 1.00 | 1.00 | 0.53 | 1.00 | 1.00 | 1.00 | 1.00 |
| UMNS | **0.00** | 0.14 | **0.00** | 0.53 | 0.99 | 1.00 | 1.00 |
| RVS CR | **0.00** | **0.00** | **0.00** | **0.01** | 0.53 | 1.00 | 1.00 |
| fixed CR | **0.00** | **0.00** | **0.00** | **0.00** | **0.00** | 0.53 | 0.98 |
| SVS CR | **0.00** | **0.00** | **0.00** | **0.00** | **0.00** | **0.02** | 0.53 |

| <c_II_(256 s)> | no stim | PPMS | CMNS | UMNS | RVS CR | fixed CR | SVS CR |
| --- | --- | --- | --- | --- | --- | --- | --- |
| no stim | 0.53 | **0.00** | 1.00 | **0.00** | **0.00** | **0.00** | **0.00** |
| PPMS | 1.00 | 0.53 | 1.00 | 0.12 | **0.00** | **0.00** | **0.00** |
| CMNS | **0.00** | **0.00** | 0.53 | **0.00** | **0.00** | **0.00** | **0.00** |
| UMNS | 1.00 | 0.89 | 1.00 | 0.53 | **0.01** | **0.00** | **0.00** |
| RVS CR | 1.00 | 1.00 | 1.00 | 0.99 | 0.53 | **0.03** | **0.00** |
| fixed CR | 1.00 | 1.00 | 1.00 | 1.00 | 0.97 | 0.53 | 0.09 |
| SVS CR | 1.00 | 1.00 | 1.00 | 1.00 | 1.00 | 0.92 | 0.53 |

**Supplementary Table 4**: *p*-values belonging to the stimulation intensity *K* = 0.25 by comparing the boxplots in **Figures 2,3** with **4** obtained by the left-sided Wilcoxon rank sum test for different stimulation approaches. *p*-values of smaller values which are statistically significant are highlighted in bold (significance level α = 0.05), indicating e.g. for the CMNS protocol applied at *K*=0.25 the following statistically significant relations *C_av_* (*t* = 128 s) **>** *C_av_* (*t* = 256 s) and *R_av_* (*t* = 128 s) **<** *R_av_* (*t* = 256 s).

| $C_{av}$ | x1=  no stim  *t*=128 s | x2=  no stim  *t*=256 s | x1=  PPMS  *t*=128 s | x2=  PPMS  *t*=256 s | x1=  CMNS  *t*=128 s | x2=  CMNS  *t*=256 s |
| --- | --- | --- | --- | --- | --- | --- |
| x1 | 0.53 | 0.80 | 0.53 | 0.67 | 0.53 | 1.00 |
| x2 | 0.22 | 0.53 | 0.35 | 0.53 | **0.00** | 0.53 |

| $C_{av}$ | x1=  UMNS  *t*=128 s | x2=  UMNS  *t*=256 s | x1=  RVS  *t*=128 s | x2=  RVS  *t*=256 s | x1=  fixed  *t*=128 s | x2=  fixed  *t*=256 s | x1=  SVS  *t*=128 s | x2=  SVS  *t*=256 s |
| --- | --- | --- | --- | --- | --- | --- | --- | --- |
| x1 | 0.53 | **0.01** | 0.53 | 0.08 | 0.53 | 0.53 | 0.53 | **0.00** |
| x2 | 1.00 | 0.53 | 0.93 | 0.53 | 0.50 | 0.53 | 1.00 | 0.53 |

| $R_{av}$ | x1=  no stim  *t*=128 s | x2=  no stim  *t*=256 s | x1=  PPMS  *t*=128 s | x2=  PPMS  *t*=256 s | x1=  CMNS  *t*=128 s | x2=  CMNS  *t*=256 s |
| --- | --- | --- | --- | --- | --- | --- |
| x1 | 0.53 | 0.45 | 0.53 | 0.55 | 0.53 | **0.00** |
| x2 | 0.58 | 0.53 | 0.47 | 0.53 | 1.00 | 0.53 |

| $R_{av}$ | x1=  UMNS  *t*=128 s | x2=  UMNS  *t*=256 s | x1=  RVS  *t*=128 s | x2=  RVS  *t*=256 s | x1=  fixed  *t*=128 s | x2=  fixed  *t*=256 s | x1=  SVS  *t*=128 s | x2=  SVS  *t*=256 s |
| --- | --- | --- | --- | --- | --- | --- | --- | --- |
| x1 | 0.53 | 0.53 | 0.53 | **0.00** | 0.53 | 0.15 | 0.53 | **0.00** |
| x2 | 0.50 | 0.53 | 1.00 | 0.53 | 0.86 | 0.53 | 1.00 | 0.53 |

**Supplementary Table 5**: *p*-values belonging to the stimulation intensity *K* = 0.10 in **Figure 10** obtained by the left-sided Wilcoxon rank sum test for different stimulation approaches. *p*-values of smaller values which are statistically significant are highlighted in bold (significance level α = 0.05).

| C_av_ (128 s) | no stim | PPMS | CMNS | UMNS | RVS CR | fixed CR | SVS CR |
| --- | --- | --- | --- | --- | --- | --- | --- |
| no stim | 0.53 | 0.10 | **0.00** | 1.00 | 1.00 | **0.00** | **0.00** |
| PPMS | 0.91 | 0.53 | **0.00** | 1.00 | 1.00 | **0.00** | **0.00** |
| CMNS | 1.00 | 1.00 | 0.53 | 1.00 | 1.00 | 0.89 | 0.98 |
| UMNS | **0.00** | **0.00** | **0.00** | 0.53 | 0.90 | **0.00** | **0.00** |
| RVS CR | **0.00** | **0.00** | **0.00** | 0.11 | 0.53 | **0.00** | **0.00** |
| fixed CR | 1.00 | 1.00 | 0.12 | 1.00 | 1.00 | 0.53 | 0.72 |
| SVS CR | 1.00 | 1.00 | **0.03** | 1.00 | 1.00 | 0.30 | 0.53 |

| R_av_ (128 s) | no stim | PPMS | CMNS | UMNS | RVS CR | fixed CR | SVS CR |
| --- | --- | --- | --- | --- | --- | --- | --- |
| no stim | 0.53 | 1.00 | 0.55 | 1.00 | 1.00 | 0.65 | 0.93 |
| PPMS | **0.00** | 0.53 | **0.00** | 1.00 | 1.00 | **0.00** | **0.00** |
| CMNS | 0.47 | 1.00 | 0.53 | 1.00 | 1.00 | 0.63 | 0.67 |
| UMNS | **0.00** | **0.00** | **0.00** | 0.53 | 0.93 | **0.00** | **0.00** |
| RVS CR | **0.00** | **0.00** | **0.00** | 0.08 | 0.53 | **0.00** | **0.00** |
| fixed CR | 0.37 | 1.00 | 0.40 | 1.00 | 1.00 | 0.53 | 0.80 |
| SVS CR | 0.09 | 1.00 | 0.35 | 1.00 | 1.00 | 0.22 | 0.53 |

| C_av_ (256 s) | no stim | PPMS | CMNS | UMNS | RVS CR | fixed CR | SVS CR |
| --- | --- | --- | --- | --- | --- | --- | --- |
| no stim | 0.53 | 0.42 | **0.00** | 0.76 | 0.97 | **0.00** | **0.02** |
| PPMS | 0.60 | 0.53 | **0.00** | 0.76 | 0.98 | **0.01** | 0.08 |
| CMNS | 1.00 | 1.00 | 0.53 | 1.00 | 1.00 | 0.70 | 0.95 |
| UMNS | 0.26 | 0.26 | **0.00** | 0.53 | 0.95 | **0.01** | **0.03** |
| RVS CR | **0.04** | **0.03** | **0.00** | 0.06 | 0.53 | **0.00** | **0.01** |
| fixed CR | 1.00 | 0.99 | 0.33 | 0.99 | 1.00 | 0.53 | 0.89 |
| SVS CR | 0.98 | 0.93 | 0.06 | 0.97 | 1.00 | 0.12 | 0.53 |

| R_av_ (256 s) | no stim | PPMS | CMNS | UMNS | RVS CR | fixed CR | SVS CR |
| --- | --- | --- | --- | --- | --- | --- | --- |
| no stim | 0.53 | 0.92 | 0.14 | 0.90 | 1.00 | 0.35 | 0.35 |
| PPMS | 0.09 | 0.53 | **0.03** | 0.35 | 0.96 | 0.07 | 0.09 |
| CMNS | 0.88 | 0.97 | 0.53 | 0.98 | 1.00 | 0.74 | 0.76 |
| UMNS | 0.11 | 0.67 | **0.02** | 0.53 | 0.98 | 0.09 | 0.09 |
| RVS CR | **0.00** | 0.051 | **0.00** | **0.02** | 0.53 | **0.00** | **0.00** |
| fixed CR | 0.67 | 0.94 | 0.28 | 0.92 | 1.00 | 0.53 | 0.58 |
| SVS CR | 0.67 | 0.92 | 0.26 | 0.92 | 1.00 | 0.45 | 0.53 |

**Supplementary Table 6**: *p*-values belonging to the stimulation intensity *K* = 0.15 in **Figure 10** obtained by the left-sided Wilcoxon rank sum test for different stimulation approaches. *p*-values of smaller values which are statistically significant are highlighted in bold (significance level α = 0.05).

| C_av_ (128 s) | no stim | PPMS | CMNS | UMNS | RVS CR | fixed CR | SVS CR |
| --- | --- | --- | --- | --- | --- | --- | --- |
| no stim | 0.53 | 1.00 | **0.00** | 1.00 | 1.00 | 1.00 | 1.00 |
| PPMS | **0.00** | 0.53 | **0.00** | 1.00 | 1.00 | 0.74 | 0.55 |
| CMNS | 1.00 | 1.00 | 0.53 | 1.00 | 1.00 | 1.00 | 1.00 |
| UMNS | **0.00** | **0.01** | **0.00** | 0.53 | 1.00 | 0.20 | 0.37 |
| RVS CR | **0.00** | **0.00** | **0.00** | **0.00** | 0.53 | **0.01** | 0.37 |
| fixed CR | **0.00** | 0.28 | **0.00** | 0.82 | 1.00 | 0.53 | 0.65 |
| SVS CR | **0.00** | 0.47 | **0.00** | 0.65 | 0.65 | 0.37 | 0.53 |

| R_av_ (128 s) | no stim | PPMS | CMNS | UMNS | RVS CR | fixed CR | SVS CR |
| --- | --- | --- | --- | --- | --- | --- | --- |
| no stim | 0.53 | 1.00 | 1.00 | 1.00 | 1.00 | 1.00 | 1.00 |
| PPMS | **0.00** | 0.53 | **0.00** | 0.91 | 1.00 | 0.80 | 0.63 |
| CMNS | **0.00** | 1.00 | 0.53 | 1.00 | 1.00 | 1.00 | 1.00 |
| UMNS | **0.00** | 0.10 | **0.00** | 0.53 | 1.00 | 0.85 | 0.47 |
| RVS CR | **0.00** | **0.00** | **0.00** | **0.00** | 0.53 | **0.04** | 0.37 |
| fixed CR | **0.00** | 0.22 | **0.00** | 0.17 | 0.97 | 0.53 | 0.47 |
| SVS CR | **0.00** | 0.40 | **0.00** | 0.55 | 0.65 | 0.55 | 0.53 |

| C_av_ (256 s) | no stim | PPMS | CMNS | UMNS | RVS CR | fixed CR | SVS CR |
| --- | --- | --- | --- | --- | --- | --- | --- |
| no stim | 0.53 | 1.00 | **0.00** | 1.00 | 1.00 | 0.98 | 0.93 |
| PPMS | **0.00** | 0.53 | **0.00** | 0.09 | 0.93 | 0.40 | 0.35 |
| CMNS | 1.00 | 1.00 | 0.53 | 1.00 | 1.00 | 1.00 | 1.00 |
| UMNS | **0.01** | 0.92 | **0.00** | 0.53 | 1.00 | 0.85 | 0.65 |
| RVS CR | **0.00** | 0.08 | **0.00** | **0.00** | 0.53 | 0.07 | 0.18 |
| fixed CR | **0.03** | 0.63 | **0.00** | 0.17 | 0.94 | 0.53 | 0.60 |
| SVS CR | 0.08 | 0.67 | **0.00** | 0.37 | 0.83 | 0.42 | 0.53 |

| R_av_ (256 s) | no stim | PPMS | CMNS | UMNS | RVS CR | fixed CR | SVS CR |
| --- | --- | --- | --- | --- | --- | --- | --- |
| no stim | 0.53 | 1.00 | 0.15 | 1.00 | 1.00 | 1.00 | 0.99 |
| PPMS | **0.00** | 0.53 | **0.00** | 0.35 | 1.00 | 0.50 | 0.47 |
| CMNS | 0.86 | 1.00 | 0.53 | 1.00 | 1.00 | 1.00 | 1.00 |
| UMNS | **0.01** | 0.67 | **0.00** | 0.53 | 0.98 | 0.58 | 0.55 |
| RVS CR | **0.00** | **0.01** | **0.00** | **0.02** | 0.53 | **0.02** | **0.02** |
| fixed CR | **0.01** | 0.53 | **0.00** | 0.45 | 0.97 | 0.53 | 0.53 |
| SVS CR | **0.01** | 0.55 | **0.00** | 0.47 | 0.98 | 0.50 | 0.53 |

**Supplementary Table 7**: *p*-values belonging to the stimulation intensity *K* = 0.20 in **Figure 10** obtained by the left-sided Wilcoxon rank sum test for different stimulation approaches. *p*-values of smaller values which are statistically significant are highlighted in bold (significance level α = 0.05).

| C_av_ (128 s) | no stim | PPMS | CMNS | UMNS | RVS CR | fixed CR | SVS CR |
| --- | --- | --- | --- | --- | --- | --- | --- |
| no stim | 0.53 | 1.00 | **0.00** | 1.00 | 1.00 | 1.00 | 1.00 |
| PPMS | **0.00** | 0.53 | **0.00** | 1.00 | 1.00 | 1.00 | 1.00 |
| CMNS | 1.00 | 1.00 | 0.53 | 1.00 | 1.00 | 1.00 | 1.00 |
| UMNS | **0.00** | **0.00** | **0.00** | 0.53 | 1.00 | 0.86 | 1.00 |
| RVS CR | **0.00** | **0.00** | **0.00** | **0.00** | 0.53 | 0.86 | 1.00 |
| fixed CR | **0.00** | **0.00** | **0.00** | 0.15 | 0.15 | 0.53 | 1.00 |
| SVS CR | **0.00** | **0.00** | **0.00** | **0.00** | **0.00** | **0.00** | 0.53 |

| R_av_ (128 s) | no stim | PPMS | CMNS | UMNS | RVS CR | fixed CR | SVS CR |
| --- | --- | --- | --- | --- | --- | --- | --- |
| no stim | 0.53 | 1.00 | 1.00 | 1.00 | 1.00 | 1.00 | 1.00 |
| PPMS | **0.00** | 0.53 | **0.00** | 1.00 | 1.00 | 1.00 | 1.00 |
| CMNS | **0.00** | 1.00 | 0.53 | 1.00 | 1.00 | 1.00 | 1.00 |
| UMNS | **0.00** | **0.00** | **0.00** | 0.53 | 1.00 | 0.99 | 1.00 |
| RVS CR | **0.00** | **0.00** | **0.00** | **0.00** | 0.53 | 0.70 | 1.00 |
| fixed CR | **0.00** | **0.00** | **0.00** | **0.01** | 0.33 | 0.53 | 1.00 |
| SVS CR | **0.00** | **0.00** | **0.00** | **0.00** | **0.00** | **0.00** | 0.53 |

| C_av_ (256 s) | no stim | PPMS | CMNS | UMNS | RVS CR | fixed CR | SVS CR |
| --- | --- | --- | --- | --- | --- | --- | --- |
| no stim | 0.53 | 1.00 | **0.00** | 1.00 | 1.00 | 1.00 | 1.00 |
| PPMS | **0.00** | 0.53 | **0.00** | 1.00 | 1.00 | 1.00 | 1.00 |
| CMNS | 1.00 | 1.00 | 0.53 | 1.00 | 1.00 | 1.00 | 1.00 |
| UMNS | **0.00** | **0.00** | **0.00** | 0.53 | 1.00 | 0.93 | 1.00 |
| RVS CR | **0.00** | **0.00** | **0.00** | **0.00** | 0.53 | 0.67 | 1.00 |
| fixed CR | **0.00** | **0.01** | **0.00** | 0.08 | 0.35 | 0.53 | 0.99 |
| SVS CR | **0.00** | **0.00** | **0.00** | **0.00** | **0.00** | **0.01** | 0.53 |

| R_av_ (256 s) | no stim | PPMS | CMNS | UMNS | RVS CR | fixed CR | SVS CR |
| --- | --- | --- | --- | --- | --- | --- | --- |
| no stim | 0.53 | 1.00 | 0.09 | 1.00 | 1.00 | 1.00 | 1.00 |
| PPMS | **0.00** | 0.53 | **0.00** | 0.99 | 1.00 | 0.98 | 1.00 |
| CMNS | 0.92 | 1.00 | 0.53 | 1.00 | 1.00 | 1.00 | 1.00 |
| UMNS | **0.00** | **0.01** | **0.00** | 0.53 | 0.55 | 0.22 | 0.76 |
| RVS CR | **0.00** | **0.00** | **0.00** | 0.47 | 0.53 | 0.47 | 1.00 |
| fixed CR | **0.00** | **0.03** | **0.00** | 0.80 | 0.55 | 0.53 | 0.98 |
| SVS CR | **0.00** | **0.00** | **0.00** | 0.26 | **0.00** | **0.02** | 0.53 |
